# Predictive Coding and Internal Error Correction in Speech Production

**DOI:** 10.1162/nol_a_00088

**Published:** 2023-03-08

**Authors:** Alex Teghipco, Kayoko Okada, Emma Murphy, Gregory Hickok

**Affiliations:** Department of Cognitive Sciences, University of California, Irvine, CA, USA; Department of Psychology, Loyola Marymount University, Los Angeles, CA, USA

**Keywords:** speech production, fMRI, internal error correction, predictive coding, internal models, imagined speech, overt speech, nonwords, taboo words, tongue twisters

## Abstract

Speech production involves the careful orchestration of sophisticated systems, yet overt speech errors rarely occur under naturalistic conditions. The present functional magnetic resonance imaging study sought neural evidence for internal error detection and correction by leveraging a tongue twister paradigm that induces the *potential* for speech errors while excluding any overt errors from analysis. Previous work using the same paradigm in the context of silently articulated and imagined speech production tasks has demonstrated forward predictive signals in auditory cortex during speech and presented suggestive evidence of internal error correction in left posterior middle temporal gyrus (pMTG) on the basis that this area tended toward showing a stronger response when potential speech errors are biased toward nonwords compared to words (Okada et al., 2018). The present study built on this prior work by attempting to replicate the forward prediction and lexicality effects in nearly twice as many participants but introduced novel stimuli designed to further tax internal error correction and detection mechanisms by biasing speech errors toward taboo words. The forward prediction effect was replicated. While no evidence was found for a significant difference in brain response as a function of lexical status of the potential speech error, biasing potential errors toward taboo words elicited significantly greater response in left pMTG than biasing errors toward (neutral) words. Other brain areas showed preferential response for taboo words as well but responded below baseline and were less likely to reflect language processing as indicated by a decoding analysis, implicating left pMTG in internal error correction.

## INTRODUCTION

Speaking is a deceptively complex task involving several computational stages: selecting words from a mental dictionary that can contain tens of thousands of entries, correctly accessing and coding the sequence of sounds that could have many possible permutations, and executing the motor commands to reproduce those sounds with coordinated movements of several independent articulators within the vocal tract (Levelt, 1989). Given the system’s complexity, the ample opportunities for error, and the fact that speech is articulated at a rate of approximately five syllables per second (Jacewicz et al., 2010), it is remarkable that the vast majority of words are accurately produced (estimates put the number at approximately 99.9%; see, e.g., Garnham et al., 1982; Levelt, 1992). How is this achieved? One possibility is that the mechanism is so exquisitely tuned that it simply makes very few coding errors. Another possibility is that coding errors occur more frequently, but are unconsciously detected and corrected internally, prior to ever being spoken (Hickok, 2012; Levelt, 1983; Nozari et al., 2011). The latter possibility is consistent with the idea from the motor control literature that the brain simulates the position and trajectory of the motor effector it is controlling, a so-called forward internal model, as a mechanism for improving the speed and accuracy of movements via predictive coding (Kawato, 1999; Shadmehr & Krakauer, 2008; Wolpert et al., 1995). Internal predictive coding may provide a mechanism to detect and correct speech errors prior to producing them (Hickok, 2012).

Although talkers can detect and correct *overtly* produced speech errors, evidence for *internal* error correction in speech is limited to inferences based on the timing of error corrections. For example, overtly realized error corrections, such as “v-horizontal,” have been argued to occur too quickly to be accomplished using overt feedback alone, which in turn suggests the existence of at least an internal mechanism to detect, if not correct errors (Nooteboom, 2005; Nozari et al., 2011). Further evidence for internal error *detection* comes from electrophysiological measures, which have identified signals that predict speech errors prior to their vocalization (Möller et al., 2007). Strong direct evidence for internal error *correction* is sparse, however.

In one fMRI study, Okada et al. (2018) reported suggestive evidence for the existence of an internal error correction mechanism. In their experiment, the authors presented participants with tongue twister sequences that were designed to bias speech errors toward either words (REEF LEECH ➔ LEAF REACH) or nonwords (WREATH LEAGUE ➔ LEATH REEG), a paradigm previously investigated by Oppenheim and Dell (2008). Behavioral research on slips of the tongue has shown that nonword errors are more rare than real word errors, the “lexical bias” effect (Baars et al., 1975; Dell, 1986; Levelt et al., 1999; Nooteboom, 2005). Several previous behavioral studies have shown that the lexical bias effect holds even when subjects do not phonate their speech and self-report their errors (Corley et al., 2011; Oppenheim & Dell, 2008, 2010). Although not the main focus of their study, a lexicality effect was reported by Okada et al. (2018) in the left posterior middle temporal gyrus (pMTG), a region implicated in lexical-level processes. That is, Okada and colleagues found greater activation in MTG when participants recited tongue twisters that were biased to produce nonword errors compared to word-errors, *even on trials in which participants responded accurately*. This is particularly interesting because no speech errors were committed on these trials—the activation differences observed in MTG reflect the potential for a word versus nonword error. The authors suggested that speech errors resulting in nonwords are more readily detectable and therefore would be more likely to be internally corrected prior to speaking. The fact that they found a clear effect of lexical status of the error bias even under conditions of accurate performance demonstrates that the system detected the distinction internally, and this could be the case only if in fact an internal error was committed and then corrected prior to accurate output. Although this is a very interesting finding, the pMTG activation in their study did not reach statistical significance corrected for multiple comparisons, and as the authors note, this work requires replication. Runnqvist et al. (2020) recently reported on a study that used a very similar design to Okada et al. (2018), and while they reported evidence of internal error correction in the cerebellum, they did not detect an effect in the cerebral cortex.

The present research builds on this prior work by attempting a direct replication of Okada et al. (2018) along with an extension to potentially render the paradigm more sensitive to detecting evidence for an internal error correction mechanism. The experiment we introduce uses the same tongue twister stimulus list and the same tasks (silent articulation and imaging) as previous fMRI work, but additionally includes a set of stimuli designed to increase error salience and therefore error detection and correction, thereby increasing our chances of observing evidence for such a process. Previous work has shown that tongue twisters with the potential to induce taboo word slips (e.g., FULL BUD BUCK FUSS) elicit significantly fewer slips than neutral tongue twisters, suggesting a higher rate of internal error detection and correction (Motley et al., 1982). We hypothesized that tongue twisters biased toward taboo word errors would elicit greater activation in pMTG lexical networks compared to non-taboo word errors. The basis for this is that detecting and correcting word-level speech errors presumably drives activity to areas critical for lexical processing, which implicates pMTG (e.g., Indefrey, 2011; Lau & Namyst, 2019), and that prior work has found suggestive evidence for internal error correction in pMTG using tongue twisters (Okada et al., 2018; a possible mechanism of this effect is explored in the Discussion section).

In addition to mapping an error correction mechanism in the brain, we aim to replicate Okada et al.’s (2018) finding that motor-driven forward predictive signals are present in auditory cortex in a speech production task. In their study, Okada et al. (2018) had participants silently recite a sequence of tongue twisters in an fMRI experiment. Two speech production conditions were included, one in which speech was articulated without phonating (silent articulation) and one in which speech production was imagined without articulation (imagined). Both conditions were matched for acoustic input (i.e., no speech input). Previous behavioral research has shown that these two tasks engage different levels of linguistic/motor planning. Imagined speech engages lexical-level processes but not lower-level phonological processes whereas silently articulating speech engages both levels of processing. Therefore, engaging motor-phonological processes should generate a forward prediction of the acoustic consequences of the executed (silent) speech, whereas engaging lexical-level processes should not. As expected, a contrast of silently articulated speech compared to imagined speech revealed activity in left inferior frontal gyrus (IFG) and premotor cortex, areas involved in speech articulation. More interestingly, they found robust activity in bilateral auditory cortex when motor articulators were engaged, but not when speech was imagined, and this activation was present even though there was no external auditory stimulation. The authors suggest that these activations reflect stronger forward predictions generated in the articulation condition compared to the imagining condition (Levelt, 1989).

The main goal of the present research is to examine a speech production mechanism that has been elusive thus far: neural evidence of internal error correction. To that end, we leverage a tongue twister paradigm that has been previously used to generate suggestive evidence of internal error correction during speech in the pMTG. Using the same stimulus set and design as this prior work, we attempt to replicate evidence for internal error correction in a much larger sample of participants. Critically, we also improve our odds of finding evidence of internal error correction by introducing additional stimuli designed to tax the error correction mechanism: tongue twisters that elicit taboo word errors. We also attempt to replicate the forward predictive signal effect reported by this previous work. Replication is particularly important since the aforementioned study was the first fMRI experiment to show evidence of forward predictive signals involving auditory cortex, a sensory region that plays an important role in speech production.

## MATERIALS AND METHODS

### Participants

Forty participants (25 females) between 18 and 40 years of age were recruited from the University of California, Irvine (UCI) community. Participants received monetary compensation for their participation. The volunteers were right-handed, native English speakers with normal or corrected-to-normal vision, no known history of neurological disease, and no contraindications for MRI. Informed consent was obtained from each participant prior to participation in the study in accordance with guidelines from the local ethics committee at UCI that approved this study. A handful of participants were excluded from analysis for excessive head motion (*N* = 2) and unanticipated scanning termination for a variety of reasons (*N* = 4; i.e., claustrophobia, equipment malfunction, excessive tardiness to the point of being unable to collect more than a single fMRI session), leaving a total of 34 participants to contribute to the results. Four participants from the group were not able to complete all nine scanning sessions but completed 89% (*N* = 2; 8 sessions), 77% (*N* = 1; 7), and 66% (*N* = 1; 6) of the fMRI sessions.

The number of participants recruited for this study was anticipated based on a power analysis of pilot data collected for four participants. The power analysis was carried out over two regions of interest (ROIs) using the fMRIpower toolbox (Mumford & Nichols, 2008). Anatomical ROIs for this analysis were selected from the Harvard–Oxford cortical atlas (Desikan et al., 2006), and adjustment for Type 1 error was made by applying a Bonferroni correction based on the number of ROIs being compared (*p* < 0.005). The power analysis indicated that approximately 40 participants were necessary to achieve 80% power for detecting a forward prediction effect within Heschl’s gyrus (effect size of 1.0623; effect sizes expressed in standard deviation units, which is analogous to Cohen’s *d*) and internal error correction effects in temporooccipital middle temporal gyrus (toMTG; effect size of 0.3815 for a lexicality effect and 0.3985 for taboo effect). We note that a smaller sample size of 34 was indicated to achieve 72% power for detecting these effects. See the Data Analysis section for more information about how these effects were measured. Temporooccipital middle temporal gyrus was used for the power analysis instead of pMTG because this anatomical area aligned more closely with the foci reported by the study we sought to replicate (Okada et al., 2018).

### Stimuli and Task

Scanning took place at the Facility for Imaging and Brain Research at UCI. Participants were scanned while they recited a set of four words (e.g., lean reed reef leach) in sync with a visual metronome. Thirty-two sets of tongue twisters used in previous experiments were employed in the current study (Oppenheim & Dell, 2010). These tongue twisters are known to behaviorally elicit a lexical bias effect. Lexical bias refers to the tendency for word errors to create a real word instead of a nonword (e.g., target word is “reef” but slips to “leaf,” is more likely than if target word is “wreath” and slips to “leath” because leath is a nonword). These stimuli were designed so that if an error occurred on the third or fourth word of each sequence, the outcome would yield either a real word error (e.g., “leaf”) or a nonword error (e.g., “leath”). In addition to these tongue twisters, we included 32 taboo tongue twisters (see Table 1 for example of stimuli). In a behavioral pilot involving 28 participants (15 females), we found that these taboo tongue twisters were effective in eliciting speech errors (non-taboo error rate = 23%, taboo word error rate = 20%).

**Table 1.** Stimulus set

Non-taboo tongue twister	Taboo tongue twister
nod mod mock knob	cod mod mock cob
mine bikes bit mice	dine bite bike dice
bike wild wise bile	dial bile bike dies
bane gave gan bait	shave bane bit shank
name make mail nag	fake name nag fail
wing bib bit whip	shine bib bit ship
six finch fill sin	ding sin sick dill
jail cheek cheap jean	dale chip chick dean
lean reed reef leech	queen reed reef queer
yore wan wok yawn	core mud mum caught
gun bulb buck gull	fun bulb buck full
singe fib fish sip	nib singe sip knit
sing hitch his sick	ditch sing sick diss
zinc niece need zest	pink niece need pest
jog mod mock job	cot mod mock cob
job rob rock jot	call rob rock cot
van match mat verve	shack match mat shave
lull nudge buck love	full bud buck fun
zing bib bit zip	ting bib bit tip
hinge fib fit hip	shin fib fit ship
sing that them zed	shing them that shed
daft gab gas dam	dab laugh lamb dan
than bunk nuzz there	jan bid bizz jar
chicks fich fizz chin	jicks finch fizz gin
king hitch his kcik	ping hitch hiss pick
nab match mat nerve	shab volt vat shot
gun bulb but gull	con grub grunt cup
pen bunk nus pair	fend bus buck fair
zing that then zed	shang then that shed
gore wan watt gone	whole gone gore hat
goon nab nap gar	food tab tuck far
nun bulb but null	pun mull miss pull

**Figure 1.**
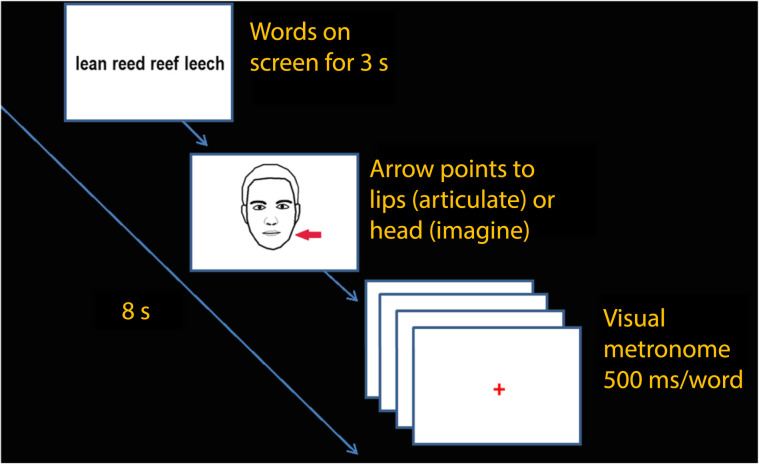
Example of a single trial. Participants were presented with a tongue twister sequence, which remained on screen for 3 s, followed by a cue to either articulate the sequence or imagine the sequence. They recited each word in sync with the visual metronome.

The present study followed the experimental procedure outlined in Okada et al. (2018). On each trial, a tongue twister phrase was visually presented on screen for 3 s, and then subjects were cued to silently articulate the sequence or imagine saying the sequence without mouth movements (see Figure 1). The presented cue was a cartoon face that remained on screen for 500 ms and contained a red arrow pointing either to the head or to the lips. An arrow pointing to the head cued the participants to imagine saying the word, and an arrow pointing to the lips cued the participants to silently articulate the words. A red fixation appeared on screen 500 ms after cue offset and served as the visual metronome, flashing at a rate of 2/s. Participants recited one word per fixation in sync with the metronome. The interstimulus interval was 500 ms. After recitation, participants indicated with a button press if they were correct or incorrect on the sequence using their left hand. Recall failure was treated as an incorrect response to ensure that only trials where rehearsal was successful (i.e., error free) would be analyzed. Participants were also instructed to indicate an incorrect response if overt production occurred accidentally. Prior to scanning, participants spent roughly 5 min (more when necessary) practicing silently reciting words in a way that minimized but did not eliminate articulatory movements. Continuous feedback was provided by lab staff during this practice period.

A single trial in the experiment was 8 s in length and there were approximately 42 trials in each session. Each session consisted of an equal number of tongue twister phrases biased to produce word errors or nonword errors and taboo errors. There were eight experimental sessions and each session consisted of approximately 14 trials of each type, which were randomly presented along with six rest trials (fixation). The study started with a high-resolution structural scan. This was followed by a short practice session of the experiment using approximately 10 trials to further familiarize subjects with the task. Scanning was conducted during the practice session to acclimatize subjects to the fMRI environment, as well as to monitor head movement and provide feedback prior to the start of the experiment. Participants also received feedback about head movement in between sessions based on qualitative assessment of the images that had been collected. The study lasted approximately 1.5 hr. Stimulus presentation and timing was controlled using PsychToolbox (Borgo et al., 2012) implemented in MATLAB (Mathworks, 2022).

### Imaging

Imaging data were collected on a 3T Siemens Prisma scanner (Siemens Medical Solutions, 2022) equipped with a 32-channel RF receiver head coil. A single T1-weighted MPRAGE sequence was acquired (matrix = 256 × 256 mm, TR = 2.3 s, TE = 2.32 ms, flip angle = 8°, size = 0.937 × 0.937 × 0.9 mm). An echo-planar imaging (EPI) pulse sequence was collected for each of the eight experimental sessions and the practice session (matrix = 100 × 100 mm, TR = 2 s, TE = 35 ms, flip angle = 90°, size = 2.4 × 2.4 × 2.4 mm, 56 slices).

### Data Analysis

Data were preprocessed and analyzed using the FMRIB’s Software Library (FSL; Jenkinson et al., 2012). First, rigid-body motion correction was performed with FSL’s intramodal motion correction tool (MCFLIRT) using the normalized correlation cost function and the middle volume as the initial template (Grabner et al., 2006). Participants with excessive head movement (>0.3 mean framewise displacement, as defined by Power et al., 2012, across sessions) were eliminated from further analysis. Mean framewise displacement among the remaining participants (*N* = 34) was relatively low (*M* = 0.15, *SD* = 0.07). Echo planar images were high pass filtered by calculating the minimal period that retains 90% of the variance in the design matrix regressors (this amounts to a roughly 0.01 hz cutoff). These images were then spatially smoothed using an isotropic 8-mm full width half maximum (FWHM) gaussian, and the anatomical image for each subject was coregistered to their middle EPI volume. Data analysis was performed with FSL’s fMRI Expert Analysis Tool (Jenkinson et al., 2012) and proceeded in three steps: (i) modeling within-session parameter estimates for events of interest using fixed effects, (ii) using these parameter estimates in a between-session analysis to model participant mean response, and (iii) using mean participant response in a between-subjects analysis to model group response using mixed effects (FSL’s FLAME1+2). Parameter estimates for each participant were transformed into standardized space using the MNI152 template (Jenkinson et al., 2002). All trials on which participants indicated making speech errors were excluded from analysis. On average, participants reported errors on 14% of trials (*SD* = 8%). Error rates were comparable across different types of tongue twister (biased toward word errors: *M* = 15.3%, *SD* = 10%; biased toward nonword errors: *M* = 15.6%, *SD* = 9.1%; biased toward taboo errors: *M* = 12.4%, *SD* = 7.8%).

Regressors for events of interest were created by convolving the predictor variables representing the time course of stimulus presentation with a gamma variate function. As in the previous experiment, regressors modeled the following experimental trial types: “Articulation: Nonword Errors,” “Articulation: Word Errors,” “Articulation: Taboo Errors,” “Imagining: Nonword Errors,” “Imagining: Word Errors,” “Imagining: Taboo Errors.” All trials on which participants reported making an incorrect response, the visual presentation of words on all trials, and the six motion parameter estimates determined during the realignment stage of preprocessing were included in the model as nuisance regressors. Regressors were used to generate parameter estimates for each condition of speech task (i.e., imagined and silently articulated) and error type (i.e., tongue twisters biased toward: nonword, word, and taboo word errors). Parameter estimates for each speech task modeled tongue twisters of all error types and parameter estimates for each error type modeled both silently articulated and imagined tongue twisters. Multiple contrasts were set up using these parameter estimates. As the primary goal of the present research was to seek evidence for internal error correction, we first tested for the lexicality effect described by Okada et al. (2018), followed by a taboo effect based on the new stimuli introduced in this study, and finally the forward prediction effect also described by Okada et al. (2018). Identical to prior work, the lexicality effect was based on the contrast between parameter estimates for the nonword and word tongue twister conditions. The same logic was extended to the novel taboo stimuli and the taboo effect was based on the contrast between taboo and word tongue twister conditions. The forward prediction effect was based on the contrast between the two speech task conditions (i.e., silently articulated vs. imagined). For each of these three contrasts an additional analysis was performed contrasting each condition in the pair against baseline fixation. The contrasts between each condition and baseline fixation were used to mask the contrast maps, allowing us to distinguish brain areas that show a significant difference in BOLD response between conditions but overall show below-baseline response.

Consistent with previous research, we expected to find taboo and lexicality effects in pMTG, and a forward predictive signal effect in auditory cortex. These hypotheses target specific regions and as such we complemented whole-brain analyses with a ROI approach that compared mean condition-level parameter estimates used in the whole brain contrast analyses within predefined anatomical areas. The same ROI analysis was carried out across and within participants, the latter of which allowed us to characterize the consistency of the effects we sought. Although the ROI analyses directly tested our hypotheses and provided complementary information to the whole-brain contrast analysis approach, we elected to place relatively greater focus on the whole-brain analysis as it allowed us to more comprehensively test which brain areas are involved in the effects we were interested in (i.e., including those areas not implicated in prior work), as well as to determine more precisely where effects within anatomical ROIs were observed. Left and right hemisphere anatomical ROIs were extracted from the Harvard–Oxford atlas. The anatomically defined pMTG area was used to test for the lexicality and taboo effects, and Heschl’s gyrus was used to test for the forward prediction effect. The ROI from the power analysis was substituted based on the results of the whole brain analyses. To foreshadow our findings, these analyses revealed a taboo effect consistent with the location of the effect reported by Okada et al. (2018), but more clearly concentrated within anatomical pMTG than toMTG. Group effects within ROIs were tested with paired *t* tests carried out across participants. Tests were additionally carried out across voxels for each participant to characterize the consistency of the effects. Minimum significance in the ROI analyses was based on a Bonferroni corrected *p*-value threshold of 0.05, and minimum significance for whole brain voxelwise testing was based on a voxelwise *p*-value threshold of 0.01 (i.e., cluster-forming *Z*-threshold of 2.6) and a GRF-based cluster size *p*-value threshold of 0.05.

In addition to the core analyses detailed above, we present post hoc analyses that give further context for some of our findings. We emphasize that these secondary analyses are incidental by separating them from the main results section. The aim of the post hoc analyses is to better functionally characterize the network of regions associated with the internal error correction effects (i.e., lexicality and taboo effects), particularly the wider network capturing regions where neural response increased during the nonword or taboo conditions relative to the word condition but overall remained below-baseline. Functions associated with this network and with each of its constituent regions were probed using the Neurosynth meta-analytic database, which associates word frequencies in the abstracts of studies with their activation foci, enabling meta-analysis of groups of studies that frequently use particular terms (Yarkoni et al., 2011). Network-level decoding was performed by computing the Pearson correlation coefficient between contrast activation maps from the current study and each of the meta-analyses that were generated for the 3,228 terms frequently used in the neuroimaging literature and embedded in Neurosynth. Briefly, performing a meta-analysis for each term involved separating all studies in Neurosynth into two groups: those that used a particular term frequently (minimum rate of 1/1,000 words which has been shown to control for incidental word usage; Yarkoni et al., 2011) and those that did not. Next, a search was performed for voxels where activity was more consistently reported in the set of studies that do frequently use the term relative to those that do not. This was accomplished by extracting the activation tables from these two groups of studies, creating contingency tables at each voxel that described whether activity was present and whether a phrase was used, and then performing a chi-square test. Due to their smaller size, regions were decoded in a slightly different way—by computing the mean posterior probability that a phrase was used within a study if activity was observed in each of a regions’ voxels. Posterior probability estimates assumed a uniform prior (i.e., all terms are equally likely to appear) and were generated for each term in Neurosynth. More complete details about how posterior probability was computed can be found in prior work (Yarkoni et al., 2011).

## RESULTS

### Core Findings

#### Internal error correction effects

Our first analysis aimed to replicate the lexicality effect described in previous work (Okada et al., 2018). Critically, *all analyses we present were restricted to error-free trials*. A group-level contrast between the nonword and word conditions of the experiment revealed no significant differences in brain response at our minimal significance thresholds (*Z* > 2.6 or *p* < 0.01; cluster corrected at *p* < 0.05). We tested for effects on the cusp of significance by avoiding cluster correction but did not find any significant differences in brain response. Lowering the cluster-forming threshold further (*Z* > 2.3) did not reveal any areas where response to nonwords was greater than response to words. Additionally, we tested whether the mean parameter estimates within the pMTG ROI differed between these two conditions but found no significant difference across participants in either the left hemisphere (parameter estimates for words: *M* = 9.59, *SD* = 41.84; parameter estimates for nonwords: *M* = 8.37, *SD* = 39.4; *t*(33) = 0.56, *p* = 0.58) or the right hemisphere (parameter estimates for words: *M* = −4.3, *SD* = 32.18; parameter estimates for nonwords: *M* = −7.5, *SD* = 28.26; *t*(33) = −1.35, *p* = 0.19). Because we failed to replicate the lexicality effect reported in previous work (i.e., no effect was observed at the group-level), we did not investigate how consistently the effect appeared within participants.

**Figure 2.**
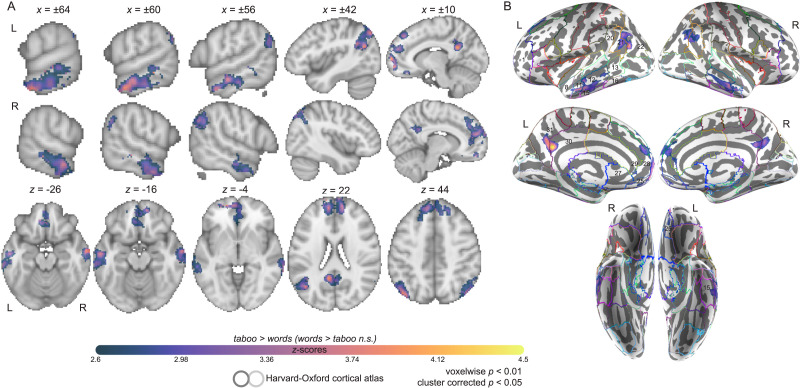
Group contrast between taboo and (neutral) word conditions. (A) select slices from the volume-based group activation map. Sagittal slices cut through peak activation observed in anterior middle temporal gyrus (*x* = ±64) and three separate peaks in posterior middle temporal gyrus (*x* = ±60, ±56). They also show the emergence of activity in superior lateral occipital cortex (*x* = ±56) and its splitting into more anterior and posterior foci, with the anterior activity intruding into angular gyrus (*x* = ±42), as well as more medial activations (*x* = ±10; i.e., posterior cingulate gyrus and frontal medial cortex). Axial slices show the same patterns: peak of activation in anterior middle temporal gyrus (*z* = −26), two peaks of activation in posterior middle temporal gyrus (*z* = −14), a third peak of activation in posterior middle temporal gyrus (*z* = −4), and two peaks of activation in superior lateral occipital cortex (*z* = 22, 44). (B) Volume-based results projected onto the fsaverage surface along with the Harvard–Oxford cortical atlas to better visualize overlap between activations and anatomical regions. Bilateral anatomical regions are shown as colored outlines and any region overlapping with activation is highlighted by an opaque superimposed number in one hemisphere that corresponds to the region’s index within the atlas. The labels for these indices are provided in Figure 4.

We next evaluated whether the novel taboo stimuli generated evidence for internal error correction by contrasting the whole-brain parameter estimates for the taboo and word conditions. As we anticipated, the taboo condition appears to have successfully increased the load on internal error detection and correction. Although no areas of the brain showed significantly greater response for the word condition than the taboo condition, relatively higher response for the taboo condition was found in a wide network that included the pMTG bilaterally (*Z* > 2.6 or *p* < 0.01, cluster corrected at *p* < 0.05 with a minimum significant cluster size of 803 voxels; see Figure 2A). A more comprehensive description of brain regions in this network was provided by registering the contrast map to anatomical areas of the Harvard–Oxford cortical atlas (Table 3). Incidental overlap between the contrast map and anatomical areas as a result of activity spilling over an anatomical boundary in a way that is inappreciable was deemphasized by focusing only on those areas in which more than 5% of voxels showed a significant difference between conditions. For visualization, both the atlas and the contrast map were projected onto the fsaverage inflated cortical surface using a recently developed procedure that implements registration fusion with advanced normalization tools to improve projection accuracy (Wu et al., 2018; Figure 2B). The network of regions that responded more strongly to the taboo than the word condition spanned the bilateral frontal poles (FPs), bilateral frontal medial cortex (FMC), bilateral superior frontal gyrus (SFG), right anterior cingulate gyrus (aCG), bilateral posterior cingulate gyrus (pCG), bilateral paracingulate gyrus (paraCG), right subcallosal cortex, bilateral precuneous cortex (preCC), bilateral anterior middle temporal gyrus (aMTG), bilateral pMTG, left posterior inferior temporal gyrus (pITG), bilateral angular gyrus (AG), and bilateral superior lateral occipital cortex (sLOC; Figure 2).

**Figure 3.**
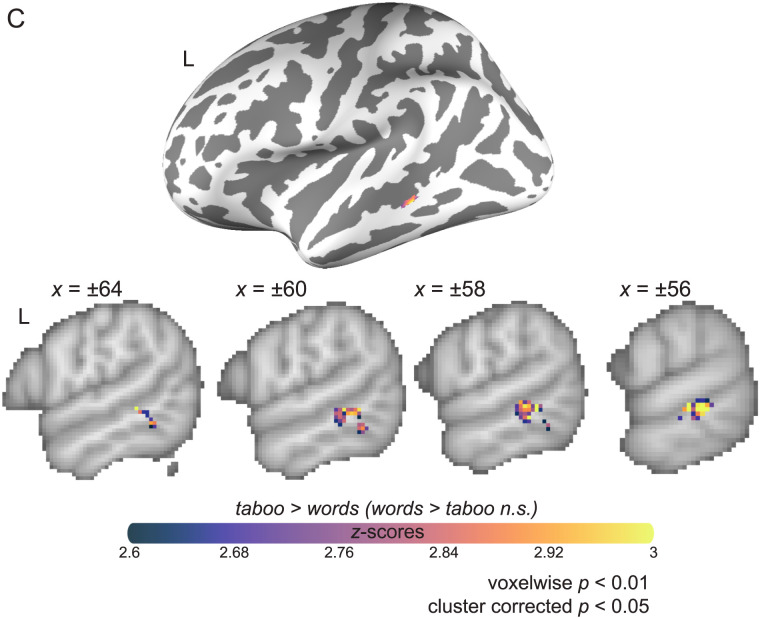
Group contrast between taboo and (neutral) word conditions: masked by significant activity during the taboo condition (i.e., taboo > baseline) and 20 voxel cluster-extent threshold is applied, revealing a large area in pMTG.

The regions that responded more strongly to the taboo than the word condition were then evaluated based on whether they showed above-baseline response during the taboo condition. Our first approach was to mask the contrast map between taboo and word conditions by the contrast map for the taboo condition (i.e., taboo > word AND taboo > baseline; both contrasts set to *Z* > 2.6 or *p* < 0.01, cluster corrected at *p* < 0.05). The resulting map produced a single large cluster and a handful of implausibly small and less meaningful clusters. A 20-voxel cluster-extent threshold was applied to the resulting map to emphasize the largest and most readily interpretable cluster in our visualization of this result in Figure 3. We emphasize that the individual maps submitted to the masking procedure were all cluster corrected themselves and the exceedingly small clusters in the masked map may reflect uninteresting differences between contrasts (e.g., noise caused by co-registration). In general, this analysis revealed that the left pMTG was by far the largest area to show significantly greater response for taboo than word conditions while also responding significantly above-baseline to the taboo condition (see Figure 3). The cluster of activity that centered on left pMTG crossed only superficially over the boundary between this area and posterior superior temporal gyrus (STG), toMTG and temporooccipital inferior temporal gyrus (ITG). We found additional clusters that peaked in pMTG below the cluster-extent threshold but note that we also found clusters below this threshold which peaked in right AG and left sLOC (Table 2).

**Table 2.** Clusters from the taboo versus word conditions contrast masked by taboo > baseline contrast

Anatomical atlas areas overlapping with cluster (% of anatomical area covered)	Cluster size (2 mm voxels)	Cohen’s *d*	Peak *z*-value	Peak *x*-coordinate	Peak *y*-coordinate	Peak *z*-coordinate	Anatomical region overlapping with peak
Left middle temporal gyrus, posterior division (7%); left superior temporal gyrus (2%); left middle temporal gyrus, temporooccipital part (2%); left inferior temporal gyrus, temporooccipital part (<1%)	140	1.78	3.88	−60	−38	−8	Left middle temporal gyrus, posterior division
Right angular gyrus (>1%); right lateral occipital cortex, superior division (>1%)	19	2.13	3.06	52	−56	46	Right angular gyrus
Left lateral occipital cortex, superior division (>1%); left angular gyrus (>1%)	15	3.56	3.67	−46	−60	46	Left lateral occipital cortex, superior division
Left lateral occipital cortex, superior division (>1%)	10	0.81	3.5	−34	−70	56	Left lateral occipital cortex, superior division
Left middle temporal gyrus, posterior division (>1%); left inferior temporal gyrus, posterior division (>1%)	4	3.9	2.84	−54	−30	−17	Left middle temporal gyrus, posterior division

**Figure 4.**
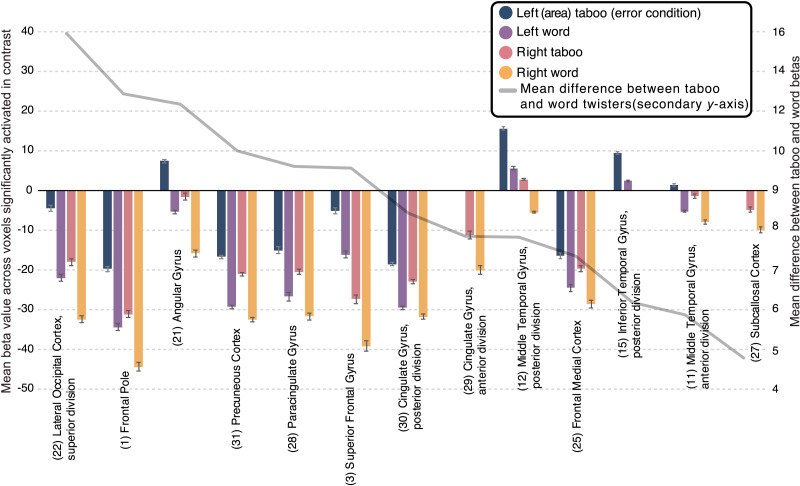
Group contrast between taboo and (neutral) word conditions: parameter estimates, or beta values for the individual conditions being contrasted, presented as an average within each anatomical region. Only voxels showing a significant effect in the contrast were included in the averages. Averages are presented separately for left hemisphere (navy and violet colored bars) and right hemisphere regions (salmon and yellow colored bars). Error bars represent standard error of the mean. Regions are organized on the *x*-axis based on descending mean difference between taboo and word parameter estimates. The magnitude of this difference is shown as a gray line that corresponds to the secondary *y*-axis.

In an additional analysis, we averaged the parameter estimates for the taboo and word conditions (i.e., taboo > baseline, word > baseline) within the portion of each anatomical area that exhibited a significant difference between these two conditions (i.e., the contrast map from Figure 2). This areal analysis was used to estimate taboo effect size and provided an additional glimpse into which areas differentially responded to the taboo and word conditions while simultaneously showing a stronger preference for baseline fixation than the task. Overall, this analysis highlighted the same group of areas as having both greater response to the taboo than word condition and above-baseline response to the taboo condition: bilateral pMTG, left AG, left pITG, and left aMTG (see Figure 4). Further, we report medium to large effect sizes (i.e., taboo vs. words) in all areas, with the pITG showing the largest effect size in the left hemisphere, and preCC showing the largest effect size in the right hemisphere (Table 3). Areas that additionally responded above baseline to the taboo condition all showed large effect sizes. No hemispheric differences were found for effect size (left: *M* = 1.17, *SD* = 0.6; right: *M* = 0.88, *SD* = 0.33; *t*(9) = 1.04, *p* = 0.32) or the spatial extent of activity within areas (left: *M* = 22%, *SD* = 16.18%; right: *M* = 19.77%, *SD* = 16.13%; *t*(9) = 1.22, *p* = 0.25), but activation maxima inside areas was typically higher in the left hemisphere (left: *M* = 3.93, *SD* = 0.37; right: *M* = 3.6, *SD* = 0.26; *t*(9) = 3.96, *p* < 0.01). Finally, we report that only a single area tended to show above-baseline response to both taboo and word conditions: the left pMTG (Figure 4). This area overlapped exclusively with the largest cluster identified when masking the taboo versus words contrast by the taboo > baseline contrast (cf. Figure 2B and Figure 3).

**Table 3.** Areal effect sizes for taboo versus word condition contrast

Atlas ROI	Left hemisphere						
	ROI size	Cohen’s *d*	Peak *z*-value	Peak *x*	Peak *y*	Peak *z*	% ROI active
(1) Frontal pole	6997	0.6	4.2	−12	66	8	14
(3) Superior frontal gyrus	3347	0.61	3.68	−10	54	26	14
(11) Middle temporal gyrus, anterior division	512	1.62	3.99	64	−6	−24	45
(12) Middle temporal gyrus, posterior division	1400	0.83	3.94	−60	−26	−8	49
(15) Inferior temporal gyrus, posterior division	1175	2.38	3.3	−60	−32	−16	14
(21) Angular gyrus	1197	1.53	4.25	−50	−56	42	28
(22) Lateral occipital cortex, superior division	5251	0.78	4.41	−36	−68	60	15
(25) Frontal medial cortex	561	0.6	3.44	−6	54	−10	51
(27) Subcallosal cortex	782	–	–	–	–	–	5
(28) Paracingulate gyrus	1720	0.71	3.73	−10	54	24	18
(29) Cingulate gyrus, anterior division	1385	–	–	–	–	–	4
(30) Cingulate gyrus, posterior division	1332	1.6	4.36	−10	−50	26	18
(31) Precuneous cortex	3047	1.62	4	−6	−54	32	11
Atlas ROI	Right hemisphere						
	ROI size	Cohen’s *d*	Peak *z*-value	Peak *x*	Peak *y*	Peak *z*	% ROI active
(1) Frontal pole	8195	0.53	3.98	12	68	22	9
(3) Superior frontal gyrus	3005	0.61	3.6	10	54	20	9
(11) Middle temporal gyrus, anterior division	472	0.79	3.45	58	−2	−26	46
(12) Middle temporal gyrus, posterior division	1374	1.49	3.88	70	−28	−4	51
(15) Inferior temporal gyrus, posterior division	1056	–	–	–	–	–	2
(21) Angular gyrus	1658	1.01	3.69	48	−54	34	18
(22) Lateral occipital cortex, superior division	5127	0.75	3.91	44	−68	44	10
(25) Frontal medial cortex	594	0.63	3.36	−2	54	−20	41
(27) Subcallosal cortex	749	0.7	3.15	2	26	−20	14
(28) Paracingulate gyrus	1650	0.71	3.59	10	52	20	26
(29) Cingulate gyrus, anterior division	1526	0.79	3.26	−2	42	−4	8
(30) Cingulate gyrus, posterior division	1393	1.12	3.55	−2	−52	32	14
(31) Precuneous cortex	3141	1.53	3.74	0	−60	38	9

**Figure 5.**
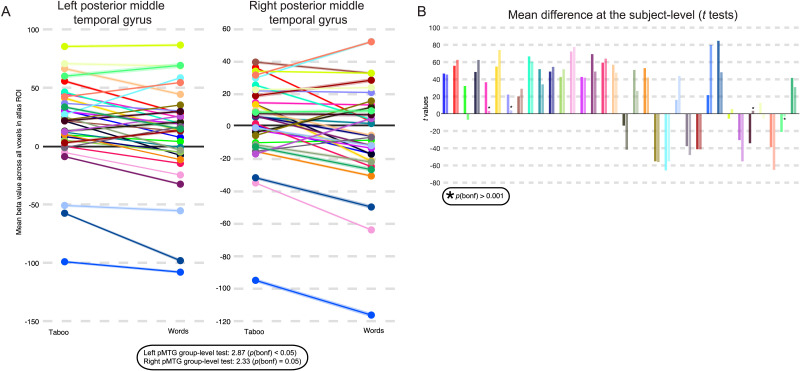
Taboo effect in posterior middle temporal gyrus (pMTG) within-participants. Anatomical ROI-based analysis of the internal error correction effect in pMTG. (A) Parameter estimates, or beta values for taboo and word conditions are averaged across all voxels of left pMTG (left) and right pMTG (right) in each subject. The shaded portions of the line plot for each subject represents standard error of the mean. Results of a paired *t* test across participants is presented below, showing significantly higher response in left pMTG to the taboo condition, and a difference between the two conditions that is on the cusp of significance for right pMTG. (B) *T* values showing magnitude and significance (*p* < 0.001; Bonferroni corrected) of parameter estimate differences between the two conditions are shown for each subject as a bar plot. Note the asterisk marks insignificant results. Each subject is assigned a color that is consistent between panels. Bars are presented in pairs such that the first and darker shaded bar of any pair represents the result of the *t* test performed on the left hemisphere ROI, and the second and lighter shaded bar represents the result of the *t* test performed over the right hemisphere ROI. Overall, 26/34 participants show a significant effect in at least one hemisphere (22 show an effect in both hemispheres).

In the preceding whole brain analyses we showed evidence for internal error correction (i.e., taboo effect) within a portion of the pMTG that our hypotheses targeted. We next carried out a more specific test for the taboo effect across all voxels of anatomically defined pMTG. Mean parameter estimates for the taboo and word conditions were extracted from pMTG voxels for each participant and a paired *t* test between conditions was performed over participants (Figure 5A). The taboo condition elicited higher parameter estimates than the word condition in the left pMTG (taboo: *M* = 18.53, *SD* = 36.77; word: *M* = 9.59, *SD* = 41.84; *t*(33) = 3.12, Bonferroni corrected *p* < 0.05). The same effect was on the cusp of significance in the right hemisphere (taboo: *M* = 2.84, *SD* = 25.66; word: *M* = −4.23, *SD* = 32.61; *t*(33) = 2.58, Bonferroni corrected *p* = 0.05). In addition, we ensured that the left hemisphere internal error correction effect was present independently during imagined (taboo: *M* = 10.24, *SD* = 19.49; word: *M* = 4.75, *SD* = 24.53; *t*(33) = 2.05, Bonferroni corrected *p* < 0.05) and silently articulated tongue twister trials (taboo: *M* = 2.84, *SD* = 25.66; word: *M* = −4.23, *SD* = 32.61; *t*(33) = 2.99, Bonferroni corrected *p* < 0.05).

Finally, we characterized the consistency of the taboo effect within anatomical pMTG by applying paired *t* tests between the taboo and word conditions within participants (Figure 5B). This analysis yielded a significant taboo effect in roughly 76% of participants (*N* = 26/34; Bonferroni-corrected *p* < 0.001). The effect occurred most often bilaterally (*N* = 22/26) and showed no hemispheric preference when it occasionally occurred in a single hemisphere (left: *N* = 2/26; right: *N* = 2/26). In four participants the effect was present, but response in pMTG was higher during baseline fixation than during the taboo condition. That is, response in pMTG was greater for the taboo than word conditions but highest for baseline fixation. In all cases where the effect was not present, we saw a significant reverse effect (*N* = 9). We considered whether portions of pMTG still showed a taboo effect in those participants who did not exhibit a mean effect across the entire pMTG by inspecting significant voxel-level differences between taboo and word conditions. No participant who showed a nonsignificant mean taboo effect presented with significant differences between taboo and word condition within portions of pMTG.

#### Forward prediction effect

**Figure 6.**
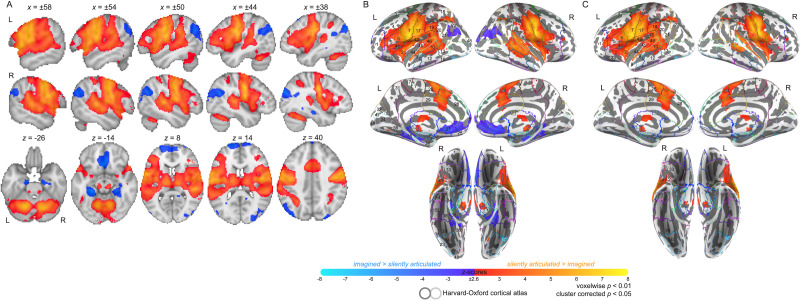
Group contrast between silently articulated and imagined speech. (A) Select slices from the volume-based group activation map. Sagittal slices cut through peak activation for the contrast in precentral and postcentral gyri (*x* = ±58) and follow Heschl’s gyrus (*x* = ±50, ±44, and ±38). Axial slices show the most ventral activations in the contrast, including the cerebellum and neighboring mesial structures (e.g., anterior paraHG and brainstem; *z* = −26), more dorsal activations in inferior temporal cortex (*z* = −14), activations along middle and superior temporal cortex (including Heschl’s gyrus; *z* = 8 and 14), and the most posterior activation in the contrast within posterior supramarginal gyrus (*z* = 40). (B) Volume-based results projected onto the fsaverage surface along with the Harvard–Oxford cortical atlas to better visualize overlap between activations and anatomical regions. Bilateral anatomical regions are shown as colored outlines and any region overlapping with activation is highlighted by an opaque superimposed number in one hemisphere that corresponds to the region’s index within the atlas. The labels for these indices are provided in Figure 7. (C) Masked by significant activity during either condition (i.e., silent articulation > baseline OR imagined > baseline), revealing that areas which showed relatively stronger response to imagined speech were deactivated during the imagined speech task.

One aim of the current study was to replicate the forward predictive signal effect described in prior work (Okada et al., 2018) by identifying brain regions that respond more strongly during silently articulated than imagined tongue twisters. To that end, we confirmed that silently articulated speech activates portions of auditory cortex more strongly than imagined speech, even though both conditions lack auditory input and do not involve overt production (*p* < 0.01 cluster corrected at *p* < 0.05; see Figure 6A–B). Overall, silently articulated tongue twisters produced greater activity than imagined tongue twisters in and around Heschl’s gyrus (HG), but also in a broad network of speech-related regions that span STG, planum temporale (PT), pMTG, toMTG, precentral gyrus, postcentral gyrus (postCG), insula, IFG, aCG, and the cerebellum (Figure 6A–B). We also found that silently articulated tongue twisters yielded greater activity in other brain regions, most of which have also been observed to activate during speech processing (Figure 6A–B). These regions were found in temporal cortex (i.e., bilateral planum polare, bilateral temporal pole), inferior temporal and neighboring portions of occipital cortex (i.e., left temporooccipital ITG, bilateral lingual gyrus, bilateral temporal occipital fusiform cortex, bilateral occipital fusiform gyrus), parietal cortex (i.e., bilateral parietal operculum, bilateral anterior and posterior supramarginal gyrus, bilateral superior parietal lobule), and frontal cortex (i.e., bilateral supplementary motor cortex, bilateral middle frontal gyrus, bilateral frontal and central operculum, bilateral SFG, left frontal orbital cortex; Figure 6A–B).

One point of difference between our results here and prior work (Okada et al., 2018) is that we also report regions that express greater activity for imagined than silently articulated word lists in several areas including bilateral anterior parahippocampal gyrus (paraHG) and posterior paraHG, bilateral posterior temporal fusiform cortex, bilateral AG, bilateral sLOC, bilateral FP, bilateral FMC, bilateral subcallosal cortex, bilateral paraCG, right preCC, and right occipital pole (OccP; Figure 6A–B).

Just as for the taboo effect, the contrast between silently articulated and imagined tongue twisters was masked by areas that showed significant above-baseline response to each of these two conditions, revealing below-baseline response almost exclusively in areas that produced significantly higher response to imagined than silently articulated tongue twisters (Figure 6C). Indeed, all areas that showed greater response to imagined tongue twisters also showed below-baseline response during the imagined condition, while only a few small areas in inferior temporal and occipital cortex that showed greater response to silently articulated tongue twisters exhibited below-baseline response during the silent articulation condition (cf. Figure 6B with 6C). The latter areas included bilateral lingual gyrus, bilateral occipital fusiform gyrus, and right OccP (cf. Figure 6B with 6C).

**Figure 7.**
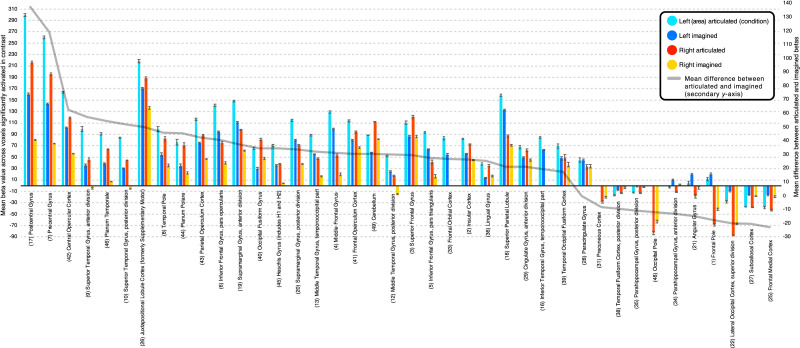
Group contrast between silently articulated and imagined speech: parameter estimates, or beta values for the individual conditions being contrasted, presented as an average within each anatomical region. Only voxels showing a significant effect in the contrast were included in the averages. Averages are presented separately for left hemisphere (blue and cyan colored bars) and right hemisphere regions (red and yellow bars). Error bars represent standard error of the mean. Regions are organized on the *x*-axis based on descending mean difference between articulated and imagined betas. The magnitude of this difference is shown as a gray line that corresponds to the secondary *y*-axis.

Areal parameter estimates for silently articulated and imagined tongue twister conditions confirmed that areas with stronger response to the silently articulated condition all showed above-baseline response (Figure 7). However, this analysis also revealed areas with stronger response to the imagined condition that showed above-baseline response, mainly left AG, left FP, and left anterior paraHG (Figure 7). Notably, parameter estimates for these three areas were relatively low for the imagined condition. The contrast between silently articulated and imagined conditions that was masked by above-baseline imagined response did not pick out voxels in these areas due to the more stringent statistical significance criteria in that analysis.

Areal effect sizes for the contrast between silently articulated and imagined conditions were large for left anterior paraHG, but small for left AG, and negligible but significant for the left FP (Table 4). For other areas which showed greater response during the imagined than silently articulated condition effect sizes were medium to large except in the right FP, bilateral sLOC, right AG, and right OccP, where effect sizes were small. The remaining areas all showed relatively greater response to silently articulated speech. A large effect was confirmed in Heschl’s gyrus bilaterally (Table 4). The second largest effect size we observed overall was in right Heschl’s gyrus and the largest was in neighboring right PT. In the left hemisphere, effect sizes were larger in portions of temporal cortex (STG, MTG, ITG, and PT) as well as postCG and central operculum than in Heschl’s gyrus (Table 4). Most areas that showed a stronger response for silently articulated word lists exhibited medium to large effect sizes. However, we report small effect sizes in bilateral temporal occipital fusiform cortex, left SFG, and bilateral aCG. Areal effect sizes were larger in the right hemisphere (left: *M* = 0.97, *SD* = 26.22; right: *M* = 1.2, *SD* = 0.75; *t*(35) = 3.56, *p* < 0.01). However, the contrast map between silently articulated and imagined tongue twisters covered a larger portion of areas in the left hemisphere (left: *M* = 43.21%, *SD* = 28.28%; right: *M* = 38.46%, *SD* = 26.22%; *t*(35) = 2.8, *p* < 0.01), and local maxima within areas was not significantly different between hemispheres (left: *M* = 5, *SD* = 0.77; right: *M* = 4.94, *SD* = 0.81; *t*(35) = 1.24, *p* = 0.22). In other words, the likelihood of finding activity in a particular area and the maximum activity observed inside that area were comparable across hemispheres, even though a greater portion of each area activated in the left hemisphere, and the smaller activations in right hemisphere areas tended to show a larger effect.

**Table 4.** Areal effect sizes for articulated versus imagined word lists

Atlas ROI	Left hemisphere							Right hemisphere						
	ROI size	Cohen’s *d*	Peak *z*-value	Peak *x*-coord	Peak *y*-coord	Peak *z*-coord	% ROI active	ROI size	Cohen’s *d*	Peak *z*-value	Peak *x*-coord	Peak *y*-coord	Peak *z*-coord	% ROI active
(1) Frontal pole	6997	−0.1	4.82	−44	48	20	19	8195	−0.46	4.36	40	36	10	7
(2) Insular cortex	1463	0.95	5.28	−36	−8	18	68	1465	1.09	5.37	34	−6	18	58
(3) Superior frontal gyrus	3347	0.36	5.3	−10	0	76	23	3005	0.79	4.79	0	28	52	13
(4) Middle frontal gyrus	3280	0.52	4.97	−48	30	34	27	3141	0.65	5.65	44	10	48	10
(5) Inferior frontal gyrus, pars triangularis	836	1.12	5.33	−48	28	12	62	745	0.56	4.4	46	34	8	28
(6) Inferior frontal gyrus, pars opercularis	915	0.91	6.02	−62	12	4	96	847	0.68	5.19	56	14	24	94
(7) Precentral gyrus	4981	1.22	6.59	−62	0	24	50	4822	1.45	7.01	40	−6	36	48
(8) Temporal Pole	2465	0.72	5.56	−56	14	−2	13	2490	0.83	4.68	58	12	2	14
(9) Superior temporal gyrus, anterior division	340	1.37	5.17	−66	0	6	47	348	1.47	5.02	64	0	8	47
(10) Superior temporal gyrus, posterior division	1019	1.93	4.72	−66	−8	4	94	1155	2.2	5.84	48	−28	8	92
(12) Middle temporal gyrus, posterior division	1400	1.39	4.06	−54	−40	−2	16	1374	2.02	4.67	50	−20	−4	25
(13) Middle temporal gyrus, temporooccipital part	1019	1.71	3.96	−54	−42	8	29	1287	1.93	5.28	60	−48	12	15
(16) Inferior temporal gyrus, temporooccipital part	808	1.35	4.83	−52	−58	−12	19	907	–	–	–	–	–	<1
(17) Postcentral gyrus	4046	1.55	6.38	−60	−4	26	36	3627	2.22	6.46	52	−6	40	35
(18) Superior parietal lobule	1731	0.9	4.72	−44	−38	46	15	1669	0.93	3.58	30	−50	46	7
(19) Supramarginal gyrus, anterior division	1128	0.77	4.93	−66	−30	26	82	959	1.64	4.85	48	−32	54	45
(20) Supramarginal gyrus, posterior division	1357	0.8	4.87	−52	−40	24	46	1516	1.04	4.91	58	−42	16	34
(21) Angular gyrus	1197	−0.35	3.83	−38	−50	44	17	1658	−0.29	4.33	42	−46	52	20
(22) Lateral occipital cortex, superior division	5251	−0.24	4.54	−28	−64	54	20	5127	−0.48	−4.24	28	−86	34	27
(25) Frontal medial cortex	561	−0.93	−3.72	−2	34	−14	25	594	−1.18	−3.93	2	34	−12	42
(26) Juxtapositional lobule cortex (formerly supplementary motor cortex)	874	0.79	5.28	−8	2	64	68	834	0.9	4.76	2	6	74	57
(27) Subcallosal cortex	782	−2.52	−4.53	−4	22	−6	48	749	−2.56	−4.45	−2	22	−4	44
(28) Paracingulate gyrus	1720	0.01	4.9	−12	14	38	41	1650	−0.01	4.41	10	16	40	38
(29) Cingulate gyrus, anterior division	1385	0.34	4.95	−12	12	38	42	1526	0.3	4.37	2	18	38	40
(31) Precuneous cortex	3047	–	–	–	–	–	0	3141	–	–	–	–	–	<1
(33) Frontal orbital cortex	1943	0.76	4.42	−50	20	−6	12	1681	–	–	–	–	–	4
(34) Parahippocampal gyrus, anterior division	709	−1.41	−4.19	−16	−10	−22	12	766	−1.59	−4.25	16	−10	−22	11
(35) Parahippocampal gyrus, posterior division	475	−0.8	−4.51	−28	−32	−12	46	378	−1.07	−3.72	26	−32	−10	39
(36) Lingual gyrus	1934	0.67	5.93	−18	−62	−12	20	2037	0.43	5.35	14	−66	−12	26
(38) Temporal fusiform cortex, posterior division	1001	−0.69	−3.92	−28	−34	−16	14	812	−0.99	−3.53	34	−20	−18	8
(39) Temporal occipital fusiform cortex	758	0.4	5.67	−22	−62	−14	29	937	0.2	4.79	20	−60	−14	22
(40) Occipital fusiform gyrus	1157	1.17	5.46	−20	−66	−14	19	1072	1.16	5.41	14	−70	−14	19
(41) Frontal operculum cortex	441	1.03	5.29	−52	14	−2	98	391	0.97	4.58	48	12	2	64
(42) Central opercular cortex	1079	1.41	6.36	−50	−8	20	90	990	2.05	6.52	42	−6	20	95
(43) Parietal operculum cortex	657	0.95	5.08	−54	−36	28	79	618	2.06	4.7	58	−32	26	46
(44) Planum polare	444	0.91	5.01	−58	6	2	32	489	1.28	5.06	64	−2	8	25
(45) Heschls gyrus (includes H1 and H2)	378	1.27	4.28	−56	−12	10	66	331	2.69	5.04	44	−24	10	82
(46) Planum temporale	626	1.41	5.09	−66	−14	12	100	530	2.96	5.71	50	−26	10	98
(48) Occipital pole	2759	–	–	–	–	–	2	2372	−0.32	5.12	14	−92	−12	25
(49) Cerebellum	11899	1.03	6.96	−20	−60	−22	22	11999	0.96	6.5	12	−64	−18	23

**Figure 8.**
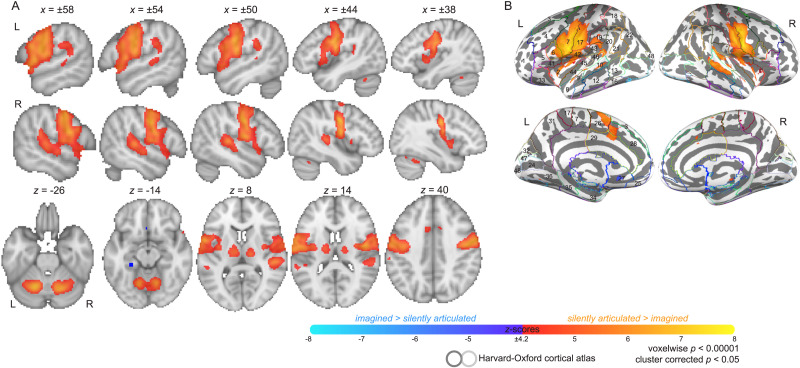
Group contrast between silently articulated and imagined speech. Minimal significance threshold from Figure 13 was increased by increments of 0.1 until the frontal-temporal cluster splintered into multiple independent clusters. (A) The results of the same contrast thresholded at *Z* > 4.2 (cluster corr., *p* < 0.05) are shown in volume-space. (B) The same maps from Figure 6A projected onto the fsaverage surface.

The minimal statistical threshold used for the contrast between silent articulation and imagined speech highlighted three large clusters. To provide a description of more meaningful clusters, we continued to increase the *Z*-threshold for this contrast map until the cluster with preferential response to silently articulated speech that covered left frontal and temporal cortex separated (*Z* > 4.2; Figure 8A–B). Critically, the resulting independent cluster within temporal cortex was just posteroventral to HG, remaining in the vicinity of auditory cortex. At this threshold, the frontal cluster peaking in left precentral gyrus extended into left supramarginal gyrus and posterior PT. The same cluster also covered left insular cortex, operculum, IFG (pars opercularis), and postCG. Activity from left frontal cortex spilled over into anterior temporal regions as well as anterior PT. Independent clusters were also found in left supplementary motor area (SMA), extending into preCC, and in left cerebellum. Anatomical overlap was remarkably similar for right hemisphere activity, but the frontal-temporal cluster remained intact, the right SMA was absent, and temporal activity was approximately closer to HG.

**Figure 9.**
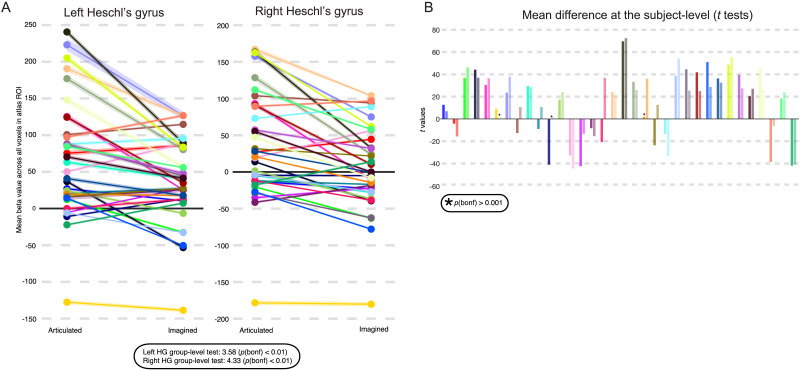
Forward prediction effects in Heschl’s gyrus within-participants. Atlas region of interest (ROI) based analysis of the forward prediction effect in Heschl’s gyrus is shown. (A) Beta values for silently articulated word lists and imagined word lists are averaged across all voxels of left and right Heschl’s gyri in each participant. The shaded portions of the line plot for each participant represents standard error of the mean. The bottom portion of the panel also reports the result of a *t* test for a group-level difference between conditions (i.e., paired *t* test across participants), which shows significantly greater response during silently articulated speech in Heschl’s gyri. (B) *T* values showing magnitude and significance (*p* < 0.001; Bonferroni corrected) of beta differences between the two conditions are shown for each participant as a bar plot. Note the asterisk marks insignificant results. Each participant is assigned a color that is consistent between panels (as well as with Figure 5). Bars representing the results of *t* tests performed on both hemisphere ROIs are presented in pairs: left (darker shaded bar) and right (lighter shaded bar). Overall, 26/34 participants show a significant effect in at least one hemisphere (20 show an effect in both hemispheres).

A test for the forward prediction effect was also carried out across all voxels of HG. Mean parameter estimates for the silently articulated and imagined conditions were extracted from HG voxels for each participant and a paired *t* test between conditions was performed over participants (Figure 9A). A forward prediction effect for mean response in HG was present at the group level, with silently articulated tongue twisters producing higher mean parameter estimates across participants than imagined tongue twisters in both the left hemisphere (articulated: *M* = 67.4, *SD* = 79.84; imagined: *M* = 36.39, *SD* = 58.1; *t*(33) = 3.62, Bonferroni-corrected *p* < 0.01) and the right hemisphere (articulated: *M* = 36.6, *SD* = 74.46; imagined: *M* = 4.15, *SD* = 59; *t*(33) = 4.52, Bonferroni-corrected *p* < 0.01).

**Figure 10.**
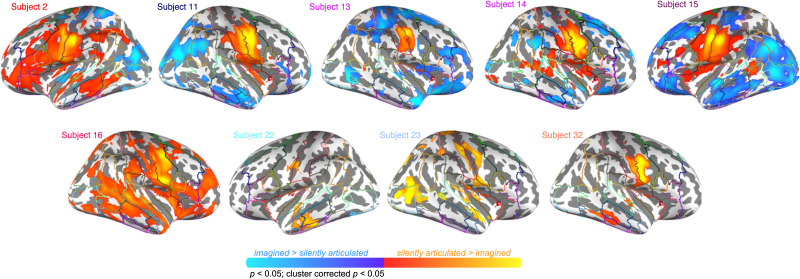
Contrast maps for example participants who show no mean forward prediction effect in Heschl’s gyrus. Maximum and minimum *z*-scores vary among participants. Participants 2, 13, 14, 15, 22, and 32 show higher response for imagined than silently articulated speech across Heschl’s gyrus bilaterally. Participant 11 shows higher response to imagined speech in left Heschl’s gyrus and no significant difference in the right hemisphere. Note, there is still higher response to silently articulated speech within Heschl’s gyrus for all participants, except Participant 13, who still shows higher response to silently articulated speech in the posterior superior temporal sulcus. Participants 16 and 23 all show higher response during silently articulated speech than imagined speech in Heschl’s gyrus bilaterally, but also present with response during silently articulated speech that is below baseline fixation. These participants’ contrast maps are masked by activity that is above baseline during silent articulation. Each participant shows higher activity for silently articulated than imagined speech in areas bordering Heschl’s gyrus.

The forward prediction effect based on mean response in HG was present in roughly 76% (*N* = 26/34) of participants (Bonferroni-corrected *p* < 0.001; Figure 9B). Most of these participants (*N* = 20/26) presented with a bilateral effect, but in one participant the effect was present only in the left hemisphere, and in five participants it was present only in the right hemisphere. Four participants that showed the effect also showed a stronger response to baseline than silent articulation. Most participants that showed no forward prediction effect in either hemisphere presented with a significant effect in the reverse direction (i.e., imagined > silently articulated across HG; *N* = 7/8), and a few participants with an established right hemisphere forward prediction effect presented with a reverse effect in the contralateral hemisphere (*N* = 4). Inspecting significant voxel-level differences between speech task conditions within participants revealed that the majority of participants that failed to show a mean effect in HG bilaterally still had voxels within that area that showed greater response to silently articulated than imagined speech (*p* < 0.05, cluster corrected *p* < 0.05; *N* = 6/8). Notably, however, this activity often peaked or at least covered areas bordering HG, including the PT, the posterior STG, the anterior STG, and the planum polare (*N* = 4/6; see participants 2, 14, 22, and 32 in Figure 10). In at least one additional participant we found evidence of stronger response to silently articulated speech, but in the posterior superior temporal sulcus (*N* = 1/8; see participant 13). By masking participant-level contrast maps (i.e., silently articulated vs. imagined) by condition-level activation maps (i.e., silently articulated vs. baseline), we also found that half of the participants that presented with a mean effect in HG but showed below-baseline response during articulated speech (bilaterally) showed some voxels in HG that responded more strongly to articulated speech and produced above-baseline response (*N* = 2/4; see participants 16 and 23 in Figure 10).

In general, we found at least one of the two effects that we sought (i.e., internal error correction and forward prediction) in 94% of participants (*N* = 32/34) and both effects in 59% of participants (*N* = 20). No significant relationship was documented between mean framewise displacement and participant-level *t* scores for contrasts between conditions (silently articulated vs. imagined speech in left HG: *r*(33) = 0.21, *p* = 0.21; silently articulated versus imagined speech in right HG: *r*(33) = 0.02, *p* = 0.93; taboo errors versus word errors in left pMTG: *r*(33) = −0.14, *p* = 0.43; taboo errors versus word errors in right pMTG: *r*(33) = −0.11, *p* = 0.53).

### Exploratory Findings

#### Comparing the default mode and taboo networks

The wider network that responded more strongly to taboo than word conditions while sometimes showing below-baseline response (referred to as TN throughout) bears a strong resemblance to the default mode network (DMN). Despite the stronger response to baseline fixation in some portions of this network, the fact that the class of the error word—the main difference between conditions—appears to be driving changes in activity entirely across the DMN is intriguing. Areas of the DMN commonly activate during mentalization and theory of mind tasks (Mars et al., 2012), suggesting that taboo words may be tapping into social cognitive systems. Here, we sought to describe the functional relationship between areas of the TN and the DMN.

**Figure 11.**
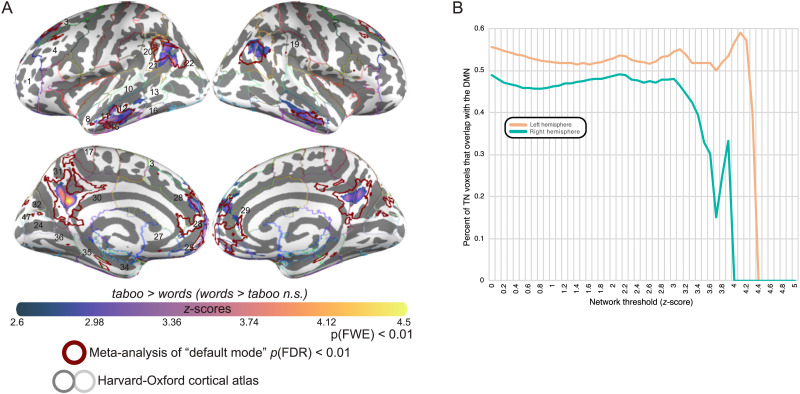
Relationship between taboo and default mode networks. Decoding the relationship between taboo and default mode networks. (A) Spatial overlap between cortical areas significantly associated with the taboo network (TN) from Figure 2 and Figure 3 and cortical areas significantly associated with the default mode network (DMN). The TN is represented by purple to yellow colors on the cortical surface and the DMN is represented by a maroon outline. The DMN is defined using the Neurosynth meta-analytic database, by analyzing activity associated with studies frequently using the term “default mode.” The resulting map represents areas significantly likely to activate across the set of studies frequently using this term (FDR corrected *p* < 0.01). (B) Percentage of TN voxels that overlap with the DMN, plotted as a function of network threshold. Overlap in the left hemisphere is plotted as a peach line and overlap in the right hemisphere is plotted as a teal line.

First, we demonstrated that areas of the TN are in fact recruited by the DMN. To define the boundaries of the DMN, we performed a meta-analysis for the phrase “default mode” in Neurosynth (FDR-corrected *p* < 0.01). The resulting meta-analytic map was found to cover canonical areas of the DMN (Mars et al., 2012): the anterior medial prefrontal cortex, pCG, AG, MTG, and paraHG. We refer to this map as the DMN throughout the results. The boundaries of the DMN were superimposed over the TN in Figure 11A, qualitatively showing that roughly the same regions activated across the two networks, but that activity was more focal in the TN and often formed a subset of each of the regions that was active in the DMN (Figure 11A). The major exception to this pattern was the paraHG, which appeared in the DMN but not in the TN. Although we saw particularly strong overlap between the two networks in the MTG, the TN contained large portions of the pMTG that did not appear in the DMN. We calculated that 45% of the voxels in the left TN and 36% of the voxels in the right TN overlapped with the DMN (Figure 11B). The finding of greater overlap in the left TN than the right was invariant to network significance thresholds (Figure 11B).

**Figure 12.**
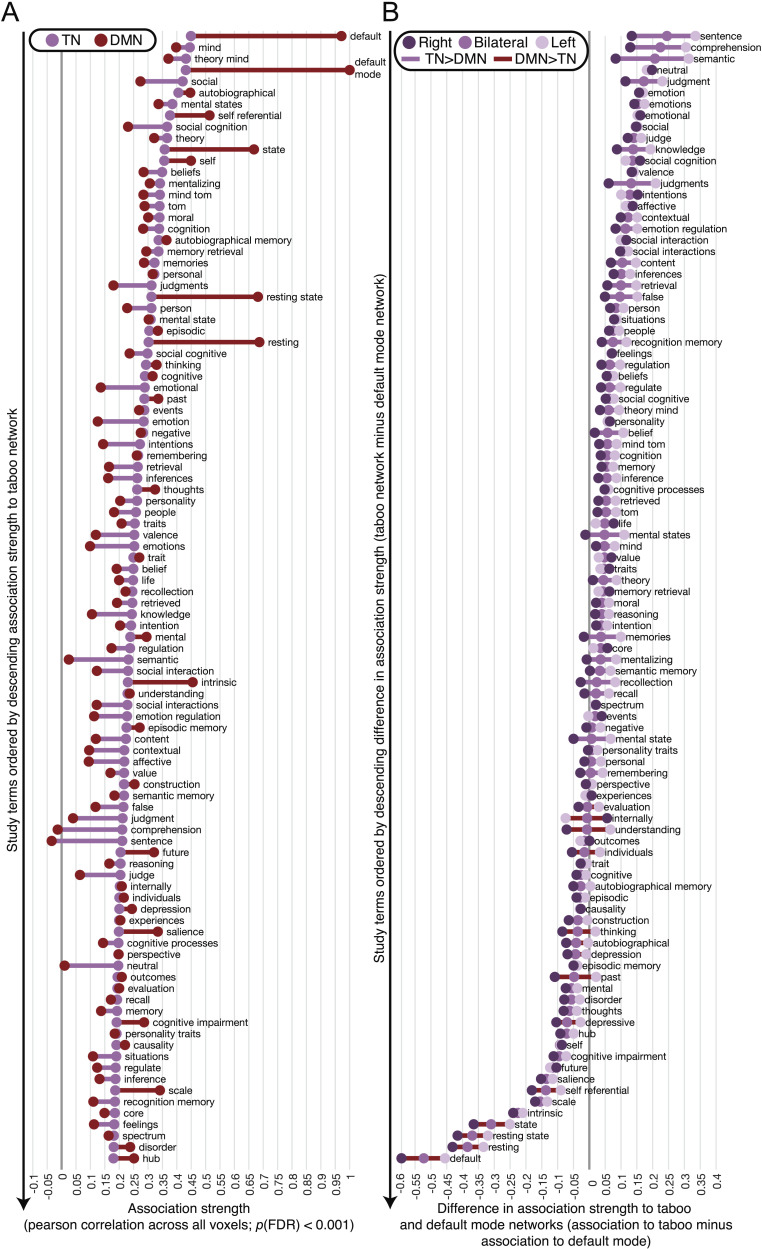
Groups of studies associated with the TN are found by correlating TN *z*-scores across the whole brain with the likelihood of seeing activity for studies frequently using each of the other terms contained in the Neurosynth database. (A) The top 100 significant correlations (FDR corrected *p* < 0.001; *r* > 0.18) are presented as purple dots and organized by descending association strength. For each of these terms, the correlation between the likelihood of seeing activity for that term and the likelihood of seeing activity for the “default mode” term (i.e., DMN) is also shown by red dots. A line is drawn between the purple and red dots to emphasize the magnitude of difference between a term and its association to the TN and DMN. The color of the lines shows which of the two networks showed the strongest association to the term. (B) Terms associated with the TN are sorted by descending difference in their association strength to the TN minus their association strength to the DMN. Associations between each term and the two networks and the differences between them are computed separately for the left hemisphere (pale purple dots), right hemisphere (deep violet dots), and bilaterally (i.e., matching the data shown in panel (A); medium violet dots). Hemisphere-dependent variability is highlighted by connecting each set of dots by a line. The color of the line shows whether the term was overall more strongly associated with the TN (violet line) or DMN (maroon line).

To better understand the relationship between the TN and the DMN, we used Neurosynth to probe for the main functional associations of the TN and then compared the extent to which the same functions were associated with the DMN. That is, we executed the same meta-analysis procedure that we used to define the DMN over the entire set of 3,228 terms frequently used in the neuroimaging literature that are embedded in Neurosynth. We then estimated the Pearson correlation coefficient between each of these meta-analytic networks and the TN. Networks were not thresholded prior to correlation because our goal was to determine purely whether the likelihood of seeing activity in a group of neuroimaging studies related to the likelihood of observing activity in the TN. However, the resulting correlation coefficients were corrected for multiple comparisons, and we focused only on the top 100 out of 2,952 significant correlations (FDR-corrected *p* < 0.001). Finally, each of the 100 networks most strongly correlated to the TN were in turn correlated to the DMN (Figure 12A). The DMN (i.e., the term “default mode”) marked the strongest relationship to the TN, explaining roughly 20% of activity in the TN and validating our early assessment of the areas that form the TN. The set of unique terms that explained the greatest amount of variance in TN activity (*r* > 0.35, *r*^2^ > 0.12) additionally included “theory of mind,” “mentalizing,” “social” [processing], “social cognition,” “autobiographical memory,” “self-referential,” and “beliefs.” In this set, associations were higher to the DMN only for the following terms: “default mode,” “self-referential.” and “autobiographical memory.” For the other terms in the set, association to the TN was on average modestly higher (mean difference in *r* = 0.07, standard error of the mean = 0.01), except for the terms “social” [processing] (DMN *r* = 0.27, TN *r* = 0.42) and “social cognition” (DMN *r* = 0.23, TN *r* = 0.36), which showed a difference that was about twice that of the mean.

A host of other terms were overall significantly associated with the TN as well. Broadly, these covered other memory systems (e.g., “episodic,” “semantic memory”), generic memory processes (e.g., “retrieval,” “recall,” “remembering,” “recollection”), emotional processes and stimuli (e.g., “emotions,” “affective,” “valence,” “emotional regulation”), references to resting state (e.g., “intrinsic,” “resting”), other social cognitive processes (e.g., “social interaction,” “inference,” “intentions,” “morals,” “personality,” “future”), decision-making (e.g., “judgments,” “value”), language (e.g., “semantic,” “sentence, “comprehension”; note semantic memory does not show a strong difference in association strength to the TN and the DMN), and disorders (e.g., “depression,” “spectrum”).

Across all terms that more strongly associated with the TN than the DMN, the language-related category of terms (e.g., semantics and sentence comprehension) showed the largest difference in association strength (i.e., between TN and DMN), followed by emotion processes and social cognition (see the bilateral plots in Figure 12B, which show the terms from Figure 12A organized by association difference). Of the terms with stronger association to the DMN, resting state terms and self-reference showed some of the largest differences with the TN. When we decomposed differences in association to the DMN and TN by hemisphere, we found that the high association difference for language-related terms was being entirely driven by activity in the left hemisphere, whereas the association difference for resting state terms and self-reference was moderately higher in the right hemisphere (Figure 12B).

#### Decoding regions of the taboo network

**Figure 13.**
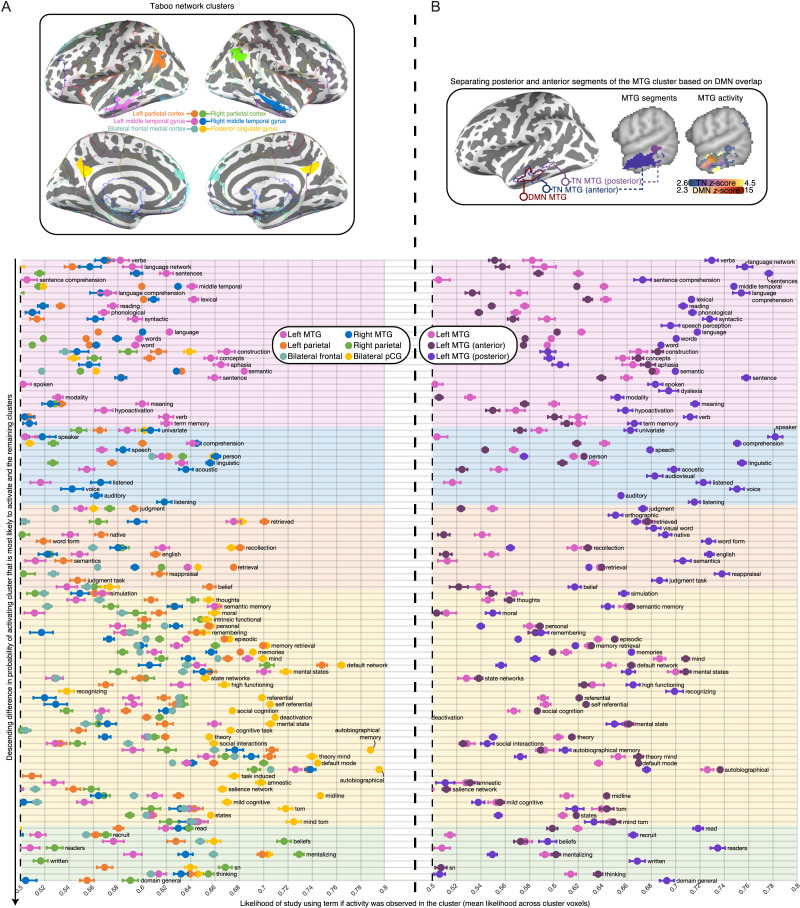
Decoding clusters of the TN. (A) The association between clusters in the TN from Figure 2A and groups of different studies in Neurosynth is found by computing the probability of studies in the database using a specific term when activity is reported in each of the voxels belonging to a cluster, then taking the mean of these probabilities across voxels of the cluster. (Top) Clusters in the TN by color; clusters are defined in volume space but projected onto the cortical surface to facilitate visualization. (Bottom) Mean probabilities for each cluster in a set of terms that has a greater than 65% mean probability of activating voxels in at least one of the six TN clusters from this panel, or two additional TN clusters from panel (B). The terms are presented in groups (by background color) based on which cluster was associated with the highest mean probability of using a term. Terms are further organized within-group by the difference in probability for the most likely cluster associated with a term, and the mean probability of the remaining clusters. Bars around each point represent standard error of the mean across voxels. Clusters with ambiguous mean probabilities are not shown (<50%). (B) Two segments of the left MTG cluster from panel (A). (Top) Left MTG cluster from panel A segmented into a posterior area in left anatomical MTG that has no spatial overlap with the DMN, and an anterior area that has some spatial overlap with the DMN. (Bottom) Mean probabilities for the same set of terms in panel (A).

The finding that activity in the left TN more closely resembled language-related terms than activity in the left DMN was remarkable because spatial overlap between the TN and DMN was higher in the left hemisphere. This suggested that what small differences existed in the distribution of activity between the left TN and DMN were functionally relevant for language. We tested whether the largest of such differences, the segment of the left pMTG that does not show overlap with the DMN was driving the taboo network-level association with language. To do this, we separated the left MTG region in the TN into two segments based on its overlap with the DMN (see top panel of Figure 13B) and then decoded the functions of both segments using Neurosynth. We note that the portion of the pMTG in the TN that did not overlap with the DMN was nearly identical to the largest region from the contrast between taboo and word conditions that also showed above-baseline response (cf. Figure 9B with Figure 3). In the interest of better characterizing the functional contributions of each region in the TN, we decoded the other regions of the TN as well, including the unsegmented left MTG (see top panel of Figure 13A). We decoded regions by generating a map for each term in Neurosynth that captures the posterior probability that a study uses a term if activity was observed in a particular voxel, assuming a uniform prior (i.e., all terms are equally likely to appear). Mean probabilities were calculated across voxels of each region in the TN, and we presented all terms that showed at least a 65% mean likelihood of being used when activity was observed in one of the regions in the TN.

Decoding each region of the TN (Figure 13A) broadly showed that different functional categories were relatively more likely to be referenced in the context of different portions of the TN. The majority of language-related terms, including sentence comprehension and lexical-semantics were more likely to be used when there was activity in the left MTG compared to other regions of the TN (e.g., see “language network,” “reading,” “syntactic,” “phonological,” “lexical,” “word,” “words,” “sentence,” “sentences,” “concepts,” “semantic,” “meaning,” “aphasia” in Figure 13A). Auditory comprehension terms were most likely to be used when the right MTG was active (e.g., see “voice,” “speaker,” “speech,” “acoustic,” “comprehension,” “listening,” “linguistic” in Figure 9A). The DMN and social cognition, including theory of mind and self-reference were most likely to be referenced when activity was in bilateral pCG (e.g., see “thoughts,” “moral,” “personal,” “theory of mind,” “episodic memory,” “autobiographical memory,” “self-referential,” “social interactions,” “default mode” in Figure 9A). Beliefs were most likely to be referenced when either of parietal regions in the TN were active, but the right parietal region was more strongly associated with multiple beliefs. The term “mentalization,” but curiously also “read,” “readers,” and “written” were most likely to be used when the right parietal region was active as well. “Word form,” “retrieval,” “recollection,” “reappraisal,” “semantics,” “judgment tasks” and “native English” are other terms that were more likely to be used when activity was observed in the left parietal region. No terms were more likely to be used when activity was found in the bilateral frontal cortex region instead of anywhere else in the TN.

Despite the relative differences in term use probabilities between regions of the TN in different functional domains, it was overwhelmingly social cognitive and memory terms that were most likely to be used when activity was found in any region of the TN. For example, the three terms that were most likely to appear when activity was found in each of the regions of the TN (Figure 9A) were largely the same: “autobiographical” (left MTG, right MTG, left parietal, bilateral pCG, bilateral frontal cortex), “autobiographical memory” (right MTG, bilateral pCG), “theory of mind” (right MTG, left parietal, bilateral frontal cortex), “mind” (left MTG, bilateral frontal cortex), “mental states” (left MTG), “mentalizing” (right parietal), “beliefs” (right parietal), and “default network” (right parietal, bilateral pCG), and “default mode” (left parietal). However, in segmenting the MTG region we found that language-related terms were more likely to be used than social cognitive terms when the posterior segment of the left MTG region was active (Figure 13B; e.g., “sentences,” “sentence,” “language network,” “language comprehension,” “comprehension,” “linguistic,” “speaker,” “voice”). In the context of activating the posterior segment of the left MTG, the highest likelihood of using a term from the social cognitive category was 67.6% for the term “autobiographical.” This likelihood was 2.4% lower than the likelihood of using the term “semantic” (70%), 3.7% lower than the likelihood of using the term “lexical” (71.3%), 7.5% lower than using the term “comprehension” (75.1%), and 10.1% lower than using the term “sentences” (77.7%). These particular language-related terms were highlighted here on the basis that they aligned with the same terms associated with activity across the entire TN (i.e., sentence comprehension and semantics), and that they additionally implicated the posterior segment of the left MTG region in lexical processes.

Language-related terms were also more likely to be used when activity was observed in the posterior segment of the left MTG than the anterior segment of the left MTG. When the posterior segment of the left MTG was active, the likelihood of using the term “semantic” was 1.9% higher (posterior left MTG: 70%; anterior left MTG: 68.1%), the likelihood of using the term “lexical” was 8.5% higher (posterior left MTG: 71.3%; anterior left MTG: 62.8%), the likelihood of using the term “comprehension” was 13.2% higher (posterior left MTG: 75.1%; anterior left MTG: 61.9%), and the likelihood of using the term “sentences” was 19% higher (posterior left MTG: 77.7%; anterior left MTG: 58.7%). In general, the likelihood that language-related terms were used when the posterior segment of the MTG was active was strikingly high. The top percentile (>75.4%) of all probabilities that a term was used when activity was found in any region of the TN was comprised of four terms for the pCG (i.e., “autobiographical,” “autobiographical memory,” “default network”) and five terms for the posterior segment of the MTG (“linguistic,” “speaker,” “sentence,” “sentences,” “language network”). Meanwhile, social cognitive and memory terms were still most likely to be used when the anterior segment of the MTG that overlapped with the DMN was active (e.g., see “autobiographical,” “mind,” “mental states,” “theory of mind,” “default mode” in Figure 13B). Splitting the left MTG region into anterior and posterior portions typically increased the likelihood that social cognitive terms were used in the anterior portion and decreases the likelihood that they were used in the posterior portion (e.g., see “autobiographical,” “mind,” “mental states,” “theory of mind,” “default mode,” “social cognitive” in Figure 13B).

Finally, the range of language-related terms associated with the posterior segment of the left MTG was much broader than the range of terms associated with the whole left MTG. This included terms related to auditory comprehension that were more strongly associated with the right MTG than the left MTG region, but not more strongly associated with the right MTG than the posterior segment of the left MTG. Other language-related terms that followed this pattern included “visual word form,” “English,” “semantics,” and judgment task” (previously more strongly associated with the left parietal region than other regions), and “read,” “readers,” and “written” (previously more strongly associated with the right parietal region than other regions). In addition, several language-related terms were only likely to be used when activity was found in the posterior segment of the left MTG, including the terms “orthographic,” “visual word,” “audiovisual,” and “speech perception.”

Despite the strong association between language-related terms and activity in the posterior segment of the left MTG, we did document a couple of language-related terms that were more likely to be used when activity was found in the anterior segment of the left MTG than the posterior segment. These were “concepts” (posterior left MTG: 59.6%; anterior left MTG: 67%) and “semantic memory” (posterior left MTG: 64.4%; anterior left MTG: 66.3%). Notably, other regions were likely to activate these terms as well. The term “semantic memory” was about as likely to be used when the bilateral pCG was active (66.2%), and only slightly less likely to be used when the right MTG (62.7%) or the left parietal region of the TN (62.6%) were active. The term “concepts” also had a relatively moderate likelihood of being used when the left parietal region of the TN was active (61%).

## DISCUSSION

### Main Effect of Internal Error Correction in Posterior Middle Temporal Gyrus

The main goal of the present research was to evaluate neural evidence for internal error detection and correction in speech production. To that end, we have leveraged a tongue twister paradigm that has generated suggestive evidence of internal error correction during speech production in the pMTG. That is, prior research (Okada et al., 2018) had suggested that tongue twisters designed such that slips would result in nonword errors generate greater activity in pMTG than tongue twisters designed such that slips would result in word errors, even though no overt errors occurred. This is intriguing because both conditions involved successfully reciting the same real words, and the only difference between them was an increased *potential* to produce a nonword speech error. The one caveat to this finding was that the lexicality effect ultimately failed to survive cluster correction. Here, we have tested for the same lexicality effect, using the same stimuli, but in a much larger group of participants. In addition, we have introduced a new class of stimuli designed to put greater demands on internal error correction mechanisms by biasing potential speech errors toward taboo words.

The present study found no evidence of a lexicality effect. This could be because it is a subtle effect not easily detectible with fMRI. Alternatively, it could be that the lexicality effect is caused not by a word versus nonword difference in salience to the error detection system, but by the nature of the lexical activation system itself, which has been argued to be biased toward activating real words over nonwords in the first place (Nozari & Dell, 2009). On this view, we observed no lexicality effect of internal error processing because lexicality is not a feature that this system naturally monitors. We submit that our null result is most consistent with this latter possibility.

In contrast, we observed strong taboo word effect on pMTG activation (our a priori ROI). Biasing potential speech errors toward taboo words during word list recitation generated a significantly stronger response in the pMTG relative to biasing potential speech errors toward (neutral) words. Importantly, this effect was observed on correctly produced trials where no errors occurred, indicating that at some level the system detected taboo words even though they were not present either in the stimulus or the ultimately expressed utterance. Based on the psycholinguistic literature on taboo word slips, which occur at a lower frequency than non-taboo word slips, we reason that during recitation of the taboo-biased word lists, slips occurred yielding the lexical activation of a taboo word, which, due to its salience, had a higher chance of being corrected than a non-taboo word slip (see Gabitov et al., 2020, for evidence of an important relation between error detection and the brain’s salience network). This implies that among the correct trials, internal error correction occurred more frequently for taboo-biased lists than non-taboo biased lists leading to greater activation of the pMTG, an area implicated in lexical access (e.g., Gow, 2012; Indefrey, 2011; Lau & Namyst, 2019) and lexical-semantics (e.g., Baldo et al., 2013; Devereux et al., 2013).

In a whole brain contrast between taboo and non-taboo word conditions, we demonstrated that portions of pMTG exhibited the taboo effect bilaterally. A more targeted ROI analysis using anatomically defined pMTG showed a stronger effect in the left hemisphere. The taboo effect held across all voxels of left pMTG but was on the cusp of significance in right pMTG (*p* = 0.05). Within-participant testing for the effect showed that it was quite consistent, presenting in at least one hemisphere in 76% (*N* = 26/34) of participants. In the majority of cases the effect was bilateral (*N* = 22/34), suggesting that a significant group-level effect may be difficult to map in right pMTG due to individual variability in functional response.

Why should error correction result in greater activation of the pMTG? The hierarchical state feedback control model proposed by Hickok (2012) at the phonological level of speech production can be extended to the lexical level and provides a mechanistic explanation. Hickok (2012) proposes that phonological encoding is implemented in a sensorimotor-like architecture with a sensory-related system in the posterior temporal lobe that codes the phonological targets of speech acts and a motor-related system in the inferior frontal lobe that codes phonological-level motor planning codes (a “syllabary” in psycholinguistic terms) that aim to reproduce the target. Error detection is achieved by comparing the activated “sensory-phonological” target with the “motor-phonological” plan: Specifically, the activated motor-phonological plan generates a forward prediction signal (ultimately an inhibitory input) to the sensory-phonological system where a match or mismatch is detected (via a cancellation mechanism for a match or failure of cancellation for a mismatch). Note that this does not imply that axons projection from motor to sensory systems are inhibitory signals. Long-range excitatory signals are likely to activate local inhibitory interneurons that generate the inhibitory effect (Isaacson & Scanziani, 2011). In the case of a mismatch, the sensory-phonological target remains active (it is not cancelled) and sends a corrective activation to its corresponding motor-phonological plan. Thus, error correction results in greater action of the target sensory-related network. A simple computational simulation presented by Hickok (2012) confirmed the feasibility of mechanism. Although Hickok (2012) did not propose that the lexical level was organized with a similar sensorimotor-like architecture, it has recently been extended to the lexical-syntactic level by Matchin and Hickok (2020; see also Isaacson & Scanziani, 2011).

### Main Effect of Forward Predictive Signals in Auditory Cortex

A secondary aim of the current research was to replicate evidence for forward predictive signals in auditory cortex (Okada et al., 2018). We successfully replicated this effect, showing that auditory cortex does seem to receive predictive signals during speech production. That is, we found that engaging motor-phonological and lexical-level processes together during silently articulated speech generated significantly greater activity in and around auditory cortex than engaging only lexical-level processes during imagined speech. Critically, the activity we observed in auditory cortex cannot reflect auditory stimulation because no speech output was generated in either of the speech task conditions. This forward predictive signal effect was apparent at the group-level based on a whole brain contrast between speech tasks, and a ROI analysis that showed that the effect held across all voxels of anatomically defined HG in both hemispheres. Within-participant testing revealed that the forward predictive signal effect was about as consistent as the taboo effect and presented in the same proportion of participants (*N* = 26/34). However, we also found that the majority of participants who did not exhibit a mean forward predictive signal effect in HG still presented higher brain response to silently articulated speech either in portions of HG or in bordering auditory association areas, showing that there can be substantial individual variability in the precise locus of the effect.

Response around auditory cortex can be explained in the context of a mismatch error between the auditory consequence of a planned articulatory sequence that is expected by the system, and the absence of any such signal as a result of silent articulation. Mismatch error induced by altered auditory feedback has produced a similar pattern of response in auditory cortex (Tourville et al., 2008). Speech induced suppression of auditory response as documented by electrocorticographic, electroencephalographic, and magnetoencephalographic recordings has also been interpreted in the context of a similar kind of mismatch error between a forward prediction of auditory consequences of speech and actual auditory input (Ford et al., 2010; Greenlee et al., 2011; Guenther & Hickok, 2015; Ventura et al., 2009). In models of motor control, mismatch is often extended not only to the content of speech, but also to its fundamental acoustic properties (e.g., pitch; Guenther & Hickok, 2015). This explains why FMRI studies have demonstrated a similar decrease in auditory response during silently articulated speech when it is coupled with hearing another person produce the same speech stimuli (Agnew et al., 2013).

### Network Associated With the Taboo Effect

Comparison between the taboo and word condition revealed preferential response to the taboo condition in areas outside pMTG and commonly associated with the DMN, including in AG and adjacent portions of sLOC, aMTG, pCG, paraCG, SFG, and several medial frontal areas. An additional comparison between the taboo condition and baseline fixation demonstrated that most areas that showed a taboo effect were deactivated during the taboo condition. The strongest evidence for above-baseline response was found in left pMTG, although we note that an areal analysis we performed also implied above-baseline response could be found within left AG and left pITG at significance thresholds lower than those used in the present study for direct contrast analyses.

The finding that left pMTG showed a taboo effect and had the clearest above-baseline response during the taboo condition implicated this area in internal error correction above other areas that exhibited a taboo effect. It also implied that internal error correction processes are likely left lateralized. This is further supported by the aforementioned ROI analysis of pMTG that demonstrated a stronger effect in the left hemisphere. The broader network of areas that exhibited a taboo effect also showed evidence for left lateralization—maximum activation within areas was significantly higher in the left hemisphere.

Recent research has also implicated left pMTG in word–picture interference using taboo distractor words (Hansen et al., 2019). This work has also demonstrated increased activity for taboo distractors in a thalamocortical network of areas that do not appear in the taboo effect we map using tongue twisters. The functional interpretation of this thalamocortical network is consistent with our results here—left pMTG is interpreted to reflect lexical processes, whereas other areas of the thalamocortical network (e.g., aCG, thalamus, IFG) support processing the arousing properties of taboo words and deploying attention mechanisms to suppress them. That our results did not reveal areas strongly associated with attention suggests that internal correction of speech errors, including taboo speech errors, may not require intervention from attentional mechanisms.

Although we predicted a robust response in left pMTG for taboo tongue twisters, we did not expect them to generate increased, albeit below-baseline, activity across areas of the DMN. We suggest that the taboo effect tapped into a broader lexical-semantic network and that activity in the DMN was driven by the social features of taboo words. Areas of the DMN appear to show a preference for social concepts embedded in narrative stories (Huth et al., 2016) and respond more strongly to words that connect to interpersonal interactions compared to those that do not (Lin et al., 2015, 2020). Observations like these have been used to argue that the brain areas responsible for theory of mind encode intentionality in semantic space (Binder et al., 2016). In this context, that taboo words generally elicited stronger response across the DMN is less surprising. Taboo words tend to be highly versatile and convey very different intentions (positive, negative, and otherwise) depending on the context in which they are used (Vingerhoets et al., 2013). For example, work in natural language processing has demonstrated that detection of hate speech is considerably less successful, suffering from high false positive rates, when the pragmatic functions of taboo words are not explicitly modeled (Davidson et al., 2017).

### Networks Associated With Speech Tasks

Previous research (Okada et al., 2018) has demonstrated that the contrast between silently articulated and imagined speech produces activity that is right lateralized in certain regions (e.g., sensorimotor cortex). In the present study we broke this contrast apart into anatomical areas, which showed that although generally the same areas were recruited bilaterally and maximum response within areas did not differ between hemispheres, larger portions of areas in the left hemisphere were sensitive to the difference between articulated and imagined speech, while the smaller portions of areas in the right hemisphere that were sensitive to this difference presented larger effect sizes. Overall, this describes a left hemisphere network that was more sensitive to differences between silently articulated and imagined speech and a right hemisphere network with higher specificity. In fact, the largest two effect sizes we reported were in the right PT and HG, underlining the robustness of auditory response during silently articulated speech. It is worth noting too that HG was one of the few areas that broke away from the trend we described across the contrast—response in HG was substantially higher in the right hemisphere, a much larger portion of the right HG was sensitive to the contrast, and right HG also showed a larger effect size. This pattern echoed our findings at the participant level, where a forward prediction effect was typically present in HG for both hemispheres (*N* = 20/26), but when the effect was occasionally unilateral it was most often found in the right hemisphere (*N* = 5). Thus, it is possible that right auditory cortex plays a larger role in processing forward predictive models in speech.

At the group level, greater response during silently articulated than imagined speech was found in a network that closely followed recent pooled analysis of speech production experiments (Tourville et al., 2019). The core of this network was highly consistent with the prior study we sought to replicate and primarily covered areas connected to phonological and articulatory processes, including the primary sensorimotor and somatosensory cortices (i.e., precentral and postcentral gyri), aCG, SMA, the cerebellum, the IFG, the PT, the supramarginal gyrus, the insula, and the adjacent central, frontal, and parietal operculum (e.g., Hickok, 2012, 2014; Kearney & Guenther, 2019; Price, 2012; Tourville et al., 2019). As alluded to earlier, the silent articulation network also contained areas involved in auditory processing, which were HG and the planum polare, but also associative auditory areas in anterior and posterior segments of the STG (Binder et al., 1996; Kearney & Guenther, 2019). We note that the anatomical STG we used for reference included portions of the posterior superior temporal sulcus putatively involved in speech perception (Venezia et al., 2017). Stronger activity in these areas is consistent with the behavioral research this study builds on, which has demonstrated that increasing the amount of articulation in speech imagery induces higher rates of phonological speech errors that involve similarly articulated phonemes (Oppenheim & Dell, 2010). The silent articulation network also recruited areas not as closely related to speech production. For example, some of these areas are commonly associated with executive function (i.e., the intraparietal sulcus, which crosses anatomically defined superior parietal lobule, posterior supramarginal gyrus, and sLOC; Velenosi et al., 2020), orthographic processing (i.e., temporooccipital portion of ITG and neighboring fusiform areas; Price, 2012), and lexical and/or semantic processing (i.e., MTG, temporal pole, middle frontal gyrus, SFG, and an area of anatomically defined orbitofrontal cortex that maps onto the pars orbitalis portion of the IFG in atlases that make the distinction between pars orbitalis and pars triangularis; e.g., Binder et al., 2009; Price, 2012). Nevertheless, all of these areas play a role during speech production (Price, 2012; Tourville et al., 2019), and it is possible that the phonological-level processes that distinguished silently articulated speech from imagined speech had the effect of producing greater activity in areas that interface with phonological features.

Curiously, some areas responded more strongly to imagined than silently articulated speech, including paraHG, AG, sLOC, temporal fusiform cortex, OccP, and several medial frontal areas. These are many of the same areas that exhibited stronger response to taboo tongue twisters relative to word tongue twisters, and just as in the case of the taboo effect, they were all deactivated during the imagined speech condition. However, areal effect analysis hinted that left AG, FP, and anterior paraHG may contain above-baseline response at lower significance thresholds than those used in the current work for contrast analyses. Although prior research did not observe any areas associated with imagined speech (Okada et al., 2018), a similar network has been documented in auditory and visual imagery tasks (e.g., Daselaar et al., 2010; Pearson, 2019; Tian et al., 2016), and functional connectivity between the AG and the other areas of this network has been shown to increase during imagined musical performance (instrumental or vocal; Tanaka & Kirino, 2019). The AG itself has been routinely implicated in semantic, episodic, and autobiographical memory, and response in this area has been related to subjective vividness during episodic memory retrieval and encoding (Tibon et al., 2019). Similarity between areas that show up in the DMN and areas involved in various mental imagery tasks (including auditory imagery) has implied a close relationship between mental imagery and the kinds of internally directed processes that have been connected to DMN areas (Daselaar et al., 2010; Pearson, 2019). Thus, the pattern of activity that we found could simply reflect the fact that imagined speech is relatively more internally oriented than articulated speech, or perhaps that imagined articulation taps into a domain general imagery network.

Given the association between many of the areas in the imagined speech network and memory, another possibility is that imagined speech recruits memory systems to facilitate imaging the auditory consequences of simulated speech under conditions where precise prediction is more difficult. Although we have shown here that forward predictions were stronger when articulatory features were salient in speech imagery (i.e., silently articulated speech), they still appear to be generated in a weaker form when such features are impoverished or absent (i.e., imagined speech; Tian et al., 2016). Further, if the quality of forward predictions produced in this memory-driven process (Tian et al., 2016) can be substantially different, it could also explain our observation that different portions of auditory cortex can simultaneously show greater activity for silently articulated and imagined speech in a minority of participants. At least one other explanation is that the areas associated with imagined speech reflect a different process for maintaining speech images in memory. For example, some research has related activity in areas of the imagined speech network to the detail of ongoing thoughts during working memory maintenance (Sormaz et al., 2018), and other studies have implicated the paraHG in the maintenance of novel information during working memory (Schon et al., 2016). Because verbal memory taps into areas involved in speech production (Buchsbaum & D’Esposito, 2019), it may be the case that weaker access to articulatory features in imagined speech requires additional engagement in nonverbal memory systems for maintaining speech imagery. More research is necessary to adjudicate between these possibilities and we emphasize that no areas that generated greater response to imagined than articulated speech showed clear above-baseline response during the imagined speech condition.

### Decoding the Taboo Network

We have suggested that the richness of social features in taboo words—particularly intentionality—has driven activity to areas of the DMN in the taboo contrast. We have also suggested that activity in the left pMTG does not reflect these social features, in part because this area alone shows clear above-baseline response during the recitation of taboo tongue twisters. A functional distinction between pMTG and other areas of the taboo network was further supported by a meta-analysis of studies that frequently mentioned the DMN, which showed strong overlap in most areas, but not pMTG.

Our explanation for the response pattern in the TN predicted that pMTG should be associated with language-specific processes, especially at the word level, while other areas of the TN should be associated with DMN functions relevant to processing intentionality. We tested these predictions by decoding the taboo network and its constituent regions in Neurosynth (Yarkoni et al., 2011). Decoding the TN involved correlating activity that we mapped as part of the taboo effect (i.e., taboo > words) with the likelihood of finding activity in different groups of neuroimaging studies that frequently mentioned different neuroimaging terms (i.e., meta-analyses for different terms in Neurosynth). This unsurprisingly revealed that DMN studies were most strongly associated with TN activity, followed by studies on functions commonly ascribed to areas of the DMN (e.g., “theory of mind,” “mentalization,” “self-reference,” “autobiographical memory,” “social cognition”). However, activity in the TN was also related to comprehension (e.g., “comprehension,” “semantic,” “sentence”). When we correlated the same term-based meta-analyses with the likelihood of observing activity in the DMN (i.e., the earlier DMN meta-analysis), we found that comprehension terms showed by far the largest difference between association strength to the TN and the DMN. Moreover, this difference in association was primarily driven by activity in the left hemisphere, which also overlapped more extensively with the DMN. This pattern suggested that left hemisphere areas of the TN that fall outside the DMN play a more substantial role in comprehension, and therefore that left pMTG may be driving the network-level association between the TN and comprehension.

We directly tested whether the posterior portions of the left MTG cluster in the TN was more closely connected to word-level processing by decoding the TN at a regional level, decoding both the entire MTG cluster as well as the posterior and anterior portions of it that sit outside and inside of the DMN, respectively. Decoding was made to be more sensitive to the spatial distribution of activity within smaller regions by capturing the mean likelihood that a study uses a particular term (that is frequently used in neuroimaging studies) if there is activity in each voxel of a TN cluster. We found that although virtually all language-related terms were more likely to be used when there was activity in the MTG compared to other regions of the TN, seeing activity in any cluster of the TN, including the MTG, was most likely to signal engagement in “autobiographical memory,” “theory of mind,” “mentalization,” or “default mode network.” This is exactly what might have been expected based on the results of the network-level decoding. However, separating the left MTG cluster based on overlap with the DMN produced a pattern that supported word processing in the posterior segment of this region. First, we found that activity in the anterior segment of MTG was still most likely to signal DMN-related functions, whereas activity in the posterior segment was most likely to signal engagement in language processes. Further, relative to the whole MTG cluster, the likelihood of using language-related terms generally increased and the likelihood of using DMN-related terms generally decreased when the posterior segment of MTG was active. The reverse of this trend was observed in the anterior segment of MTG. Closer inspection of the terms that were most likely to be used when the posterior segment of the MTG was active revealed an association with comprehension that was broad, covering both sentence and word stimuli. Terms that were likely to be used such as “speaker,” “acoustic,” “lexical,” “meaning,” “comprehension,” “word,” “linguistic,” and “phonological” were all consistent with a role for the pMTG in lexical processing. Other terms, such as “semantic,” “sentences,” “language comprehension,” “syntactic,” and “reading” may reflect the activity of lexical processes engaged during semantic tasks. In any case, these patterns clearly showed that one specific portion of the TN—the left pMTG—was much more strongly associated with language, and at least played a relatively more significant role in lexical processing.

Regional decoding of the TN also indicated that the posterior segment of MTG was involved in semantic processes, but not necessarily semantic memory. Activity in the anterior segment of MTG was more strongly associated with concepts and semantic memory than activity in the posterior segment of MTG. Further, semantic memory was just as strongly associated with the pCG and had a remarkably weak association with the posterior segment of MTG (i.e., less than 50% likelihood of term use). The stronger association to both social cognitive terms and semantic memory in areas of the TN that happened to show stronger overlap with the DMN was consistent with our interpretation that taboo words drive response in areas of the DMN as a result of their semantic features, mainly intentionality. This interpretation aligns with a recent proposal for a componential model of semantic representation that suggests areas involved in theory of mind and mentalization are used to encode and understand words that place stronger emphasis on intentionality (Binder et al., 2016). It also aligns with a growing body of work that shows words rich in social semantic features tap into areas that overlap with the DMN (e.g., Huth et al., 2016; Lin et al., 2015, 2018, 2020; Vingerhoets et al., 2013).

### Conclusions and Limitations

In summary, the present study has provided evidence that forward predictive signals are present in auditory cortex during speech production, and that error detection and correction in speech involves left pMTG. We have shown that silently articulated speech produces greater activity than imagined speech within early auditory areas, even though no auditory input is present during silently articulated speech. We have also shown that biasing potential word errors toward taboo words rather than neutral words during word list recitation generates greater activity in left pMTG, even though no overt speech errors are produced in either condition. Although we found similar response in areas that demonstrably overlapped with the DMN as well as right pMTG, these areas appeared to respond more robustly to baseline fixation than word list recitation. Further, we have provided evidence from meta-analysis that activity in left pMTG is most likely to reflect word-level processes and by extension word-level error correction. We have hypothesized, with some support from meta-analysis, that the other areas of the taboo network are involved in processing the social semantic features of taboo words, particularly intentionality.

Nevertheless, there are several limitations to the present research. First, overt speech typically increases the amount of motion observed during scanning. Although here we used mouthed speech, it is difficult to assess the full impact motion had on the results. In general, we reported what can be considered as relatively low motion in our group of participants (e.g., Eichenbaum et al., 2021; Yang et al., 2019). We also tried to mitigate the possibility that motion had a substantial impact on our results by showing that our primary effects of interest (i.e., the ROI analyses for forward prediction and internal error correction) are not correlated with mean framewise displacement. One other concern is that overt speech has been shown to produce susceptibility artifacts. It is uncertain the extent to which such artifacts may have impinged on our results, but we note that past studies have found these artifacts to disproportionately affect insular and opercular areas (Kemeny et al., 2005), whereas the current study focuses on lateral temporal cortex. Finally, our failure to find a lexicality effect in pMTG for tongue twisters suggests that evidence for such an effect trending toward significance in prior research may reflect a false positive result. However, we suspect this is not the case and that a larger amount of data is necessary to achieve significance for such a small effect size, especially considering that our manipulation of tongue twisters to elicit a greater potential for taboo word errors did successfully produce stronger activity in pMTG, as we had hypothesized.

## ACKNOWLEDGMENTS

The authors wish to thank the individuals that participated in this research. Discretionary research funding was provided by University of California, Irvine.

## FUNDING INFORMATION

Gregory Hickok, National Institute on Deafness and Other Communication Disorders (https://dx.doi.org/10.13039/100000055), Award ID: R01 DC009659. Alex Teghipco, National Science Foundation (https://dx.doi.org/10.13039/100000001), Award ID: DGE-1321846.

## AUTHOR CONTRIBUTIONS

**Alex Teghipco**: Data curation: Lead; Formal analysis: Lead; Investigation: Lead; Methodology: Supporting; Software: Lead; Visualization: Lead; Writing – original draft: Lead; Writing – review & editing: Equal. **Kayoko Okada**: Conceptualization: Equal; Formal analysis: Supporting; Funding acquisition: Supporting; Investigation: Supporting; Methodology: Equal; Resources: Equal; Supervision: Equal; Writing – original draft: Equal; Writing – review & editing: Equal. **Emma Murphy**: Data curation: Supporting; Formal analysis: Supporting; Investigation: Supporting; Resources: Supporting. **Gregory Hickok**: Conceptualization: Lead; Funding acquisition: Lead; Methodology: Equal; Supervision: Equal; Writing – original draft: Supporting; Writing – review & editing: Equal.

## DATA AND CODE AVAILABILITY STATEMENT

Group-level contrast maps for this study can be found on neurovault (https://identifiers.org/neurovault.collection:12982). Raw data is available to download on data dryad (https://doi.org/10.7280/D1BH77). Example code for replicating meta-analyses can be found on our GitHub repository (https://github.com/alexteghipco/MetaAnalysisResources). FMRI analyses can be replicated with FMRIB’s Software Library (Version 6.0.5.1; Jenkinson et al., 2012), using parameters provided in the methods (see FSL course preparatory material for guides on how to replicate purely using GUIs). Additional parameter information that may be useful is provided along with the neurovault collection linked above as metadata. Surface visualizations can be replicated using the brainSurfer toolbox for MATLAB (https://doi.org/10.5281/zenodo.7271544). A saved GUI state that can be loaded into brainSurfer (Teghipco, 2022) to show interactive and manipulable surface renderings identical to those shown in our figures (i.e., including all thresholds, colormaps, and other visualization settings) can be found on figshare (10.6084/m9.figshare.21357525).

## TECHNICAL TERMS

Forward predictive coding: The idea that motor plans lead to predictions of sensory consequences, facilitating error detection and correction in motor control.
Internal (speech) error correction: The correction of a speech error that is committed prior to speech output.
Functional magnetic resonance imaging (fMRI): Neuroimaging method used to detect changes in blood flow and oxygen in the brain.
Lexicality effect: Stronger neural response when recitation is biased to increase the *potential* for errors to be nonwords than words.
Posterior middle temporal gyrus (pMTG): Brain region crucial for lexical processing.
Taboo effect: Stronger neural response when recitation is biased to increase the *potential* for errors to be taboo words than non-taboo words.
